# Differential impact of the ERBB receptors EGFR and ERBB2 on the initiation of precursor lesions of pancreatic ductal adenocarcinoma

**DOI:** 10.1038/s41598-020-62106-8

**Published:** 2020-03-23

**Authors:** Nora Meyers, Claude Gérard, Frédéric P. Lemaigre, Patrick Jacquemin

**Affiliations:** Université catholique de Louvain, de Duve Institute, Brussels, Belgium

**Keywords:** Pancreatic cancer, Growth factor signalling, Computational models, Gene regulatory networks, Genetic databases, Inflammation, Oncogenesis

## Abstract

Earlier diagnosis of pancreatic ductal adenocarcinoma (PDAC) requires better understanding of the mechanisms driving tumorigenesis. In this context, depletion of Epidermal Growth Factor Receptor (EGFR) is known to impair development of PDAC-initiating lesions called acinar-to-ductal metaplasia (ADM) and Pancreatic Intraepithelial Neoplasia (PanIN). In contrast, the role of v-erb-b2 erythroblastic leukemia viral oncogene homolog 2 (ERBB2), the preferred dimerization partner of EGFR, remains poorly understood. Here, using a mouse model with inactivation of *Erbb2* in pancreatic acinar cells, we found that *Erbb2* is dispensable for inflammation- and KRas^G12D^-induced development of ADM and PanIN. A mathematical model of EGFR/ERBB2-KRAS signaling, which was calibrated on mouse and human data, supported the observed roles of EGFR and ERBB2. However, this model also predicted that overexpression of ERBB2 stimulates ERBB/KRAS signaling; this prediction was validated experimentally. We conclude that EGFR and ERBB2 differentially impact ERBB signaling during PDAC tumorigenesis, and that the oncogenic potential of ERBB2 is only manifested when it is overexpressed. Therefore, the level of ERBB2, not only its mere presence, needs to be considered when designing therapies targeting ERBB signaling.

## Introduction

Pancreatic ductal adenocarcinoma (PDAC) is one of the most aggressive cancers with a 5-year survival rate of about 7%. This poor prognosis is due to resistance to therapy and late diagnosis^1^, which prompts the need for better characterization of the molecular mechanisms that promote and drive PDAC formation.

Molecular and histological studies provided evidence that pancreatic intraepithelial neoplasia (PanIN) are preinvasive lesions of PDAC. PanIN arise from acinar cells that undergo inflammation-induced acinar-to-ductal metaplasia (ADM)^2,3^, a process leading to repression of the acinar gene expression program and the acquisition of a duct-like phenotype^4,5^. PanIN are genetically characterized by the presence of several mutations in tumor suppressors and proto-oncogenes^1,6^. The most prevalent mutation is an activating mutation in the *KRAS* oncogene (*KRAS*^*G12D*^) found in more than 90% of PDAC; this mutation is considered the initiating event of pancreatic cancer, whereas mutations in other oncogenes or tumor suppressors are required for tumor progression^7^.

The tyrosine kinase receptor EGFR/ERBB1 plays an essential role in pancreatic tumorigenesis. In its absence, oncogenic KRAS^G12D^ is unable to drive PanIN development. Yet, its role seems more important in the early phases of the disease than in PDAC progression^8,9^. Other studies suggest that ERBB2, the preferred dimerization partner of EGFR^10,11^, is also involved in PDAC^12,13^. Thus, acinar overexpression of ERBB2 leads to mild pancreatic inflammation and ADM^12^, whereas expression of a mutated active form of *Erbb2* (a mutation usually not found in PDAC) in mouse embryonic pancreas accelerates the development of PanIN after birth^13^. Unlike breast cancer where *ERBB2* is frequently amplified and plays an important role in tumor progression, *ERBB2* amplification is detected at low frequency in PDAC^14^. This contrasts with the high number of PDAC cases (about 50%) that show a detectable level of ERBB2 expression, and with the high expression of ERBB2 correlating with a higher grade of cellular atypia^15^. In addition, in a mouse model of PDAC, phosphorylation of ERK, a kinase downstream of EGFR and ERBB2, correlates better with the expression of ERBB2 than with that of EGFR^6^. Altogether, these observations suggest that ERBB2 plays a role in PDAC tumorigenesis. However, the requirement of ERBB2 in this process has not been investigated.

In the present work, we used an *Erbb2* deletion mouse model and mathematical modeling to investigate the role of ERBB2 in PDAC initiation. We find that the function of ERBB2 in pancreatic tumorigenesis differs from that of EGFR and is strongly dependent on its level of expression.

## Results

### ERBB2 is dispensable for PanIN formation

**Figure 1:**
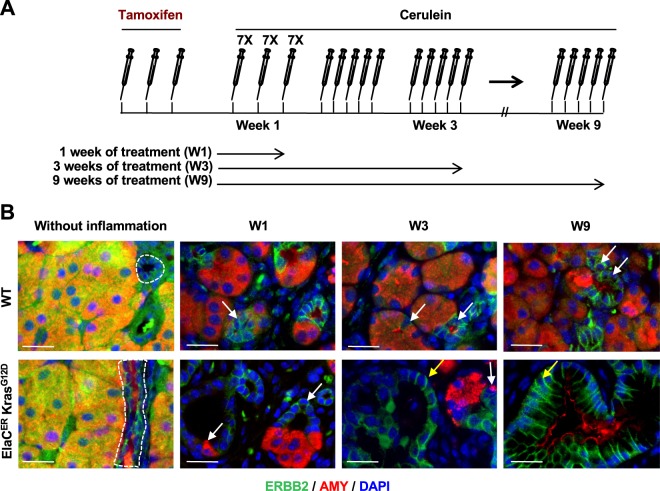
ERBB2 is expressed in ADM and PanIN. (**A**) Schedule of tamoxifen and cerulein treatments. (**B**) Immunofluorescent labeling of ERBB2 and Amylase (AMY) in wild type (WT) and ElaC^ER^ Kras^G12D^ mice without inflammation and after acute (1 week of cerulein treatment, W1) or chronic (W3 and W9) pancreatitis. ERBB2 expression is normally restricted to ductal cells (dotted lines) but is induced after inflammation in metaplastic acinar duct-like cells in WT and ElaC^ER^ Kras^G12D^ mice (white arrows), and in PanIN in ElaC^ER^ Kras^G12D^ mice (yellow arrow). DAPI is added to visualize cell nuclei. Scale bars = 50 μm.

*Egfr* deletion in pancreatic acinar cells prevents PanIN and PDAC formation in mouse models expressing oncogenic KRAS^8,9^. Since ERBB2 is the preferred dimerization partner of EGFR^10,11^, we hypothesized that ERBB2 is also important for pancreatic tumorigenesis. As a first step to address this question, we characterized the ERBB2 expression pattern in wild-type (WT) mice and in mice expressing oncogenic KRAS^G12D^ in acinar cells (ElaC^ER^ Kras^G12D^ mice), in the absence or in the presence of cerulein-induced pancreatitis (Fig. 1A,B and Supplementary Fig. S1). In WT and ElaC^ER^ Kras^G12D^ pancreas, in the absence of inflammation, ERBB2 was detected at the basolateral domains of the duct cells (dotted circle); ERBB2 labeling in acini was not membrane-associated and was considered non-specific. When WT mice received acute (W1) or chronic (W3 and W9) cerulein treatment, ERBB2 expression was ectopically induced in metaplastic acinar cells that had strongly reduced levels of amylase (late ADM; white arrows). ERBB2 expression in ElaC^ER^ Kras^G12D^ mice treated acutely with cerulein was similar to that in WT mice, with ERBB2 being detected in acinar-derived duct-like structures (white arrows). Moreover, after chronic treatment, ERBB2 expression was maintained in early and late PanIN stages (yellow arrows). Therefore, we concluded that ERBB2 is expressed in precancerous lesions of PDAC.

**Figure 2:**
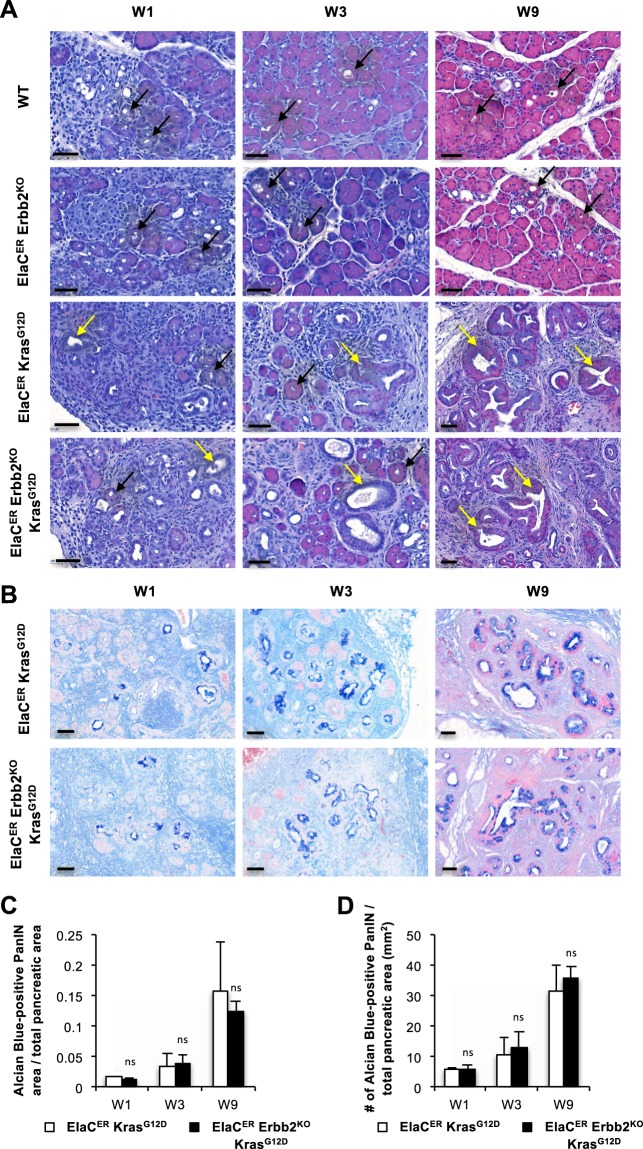
ADM and PanIN formation does not require ERBB2. (**A**) Hematoxylin and eosin staining of WT, ElaC^ER^ Erbb2^KO^, ElaC^ER^ Kras^G12D^ and ElaC^ER^ Erbb2^KO^ Kras^G12D^ mice after 1, 3 or 9 weeks of cerulein (W1, W3, W9) treatment. ADM (black arrows) and mild inflammation were present in all mice, and PanIN were detected in ElaC^ER^ Kras^G12D^ and ElaC^ER^ Kras^G12D^ Erbb2^KO^ mice. Inflammation and PanIN grade increase with the duration of the treatment. Scale bars = 50 μm. (**B**) Alcian blue staining of ElaC^ER^ Kras^G12D^ and ElaC^ER^ Erbb2^KO^ Kras^G12D^ mice after 1, 3 or 9 weeks of cerulein (W1, W3, W9) treatment. PanIN numbers increased with the duration of treatment but no significant difference was detected between the two genotypes. Scale bars = 50 μm. (**C**) The ratio of area of Alcian Blue-positive lesions to total pancreatic area increases progressively with the duration of cerulein treatment (W1, W3, W9). No significant difference is observed between the ElaC^ER^ Kras^G12D^ mice and ElaC^ER^ Erbb2^KO^ Kras^G12D^ mice. (**D**) Number (#) of Alcian Blue-positive PanIN relative to total pancreatic area (mm^2^) in ElaC^ER^ Kras^G12D^ and ElaC^ER^ Erbb2^KO^ Kras^G12D^ mice as a function of duration of cerulein treatment (W1, W3, and W9). No significant difference is observed between genotypes.

**Figure 3:**
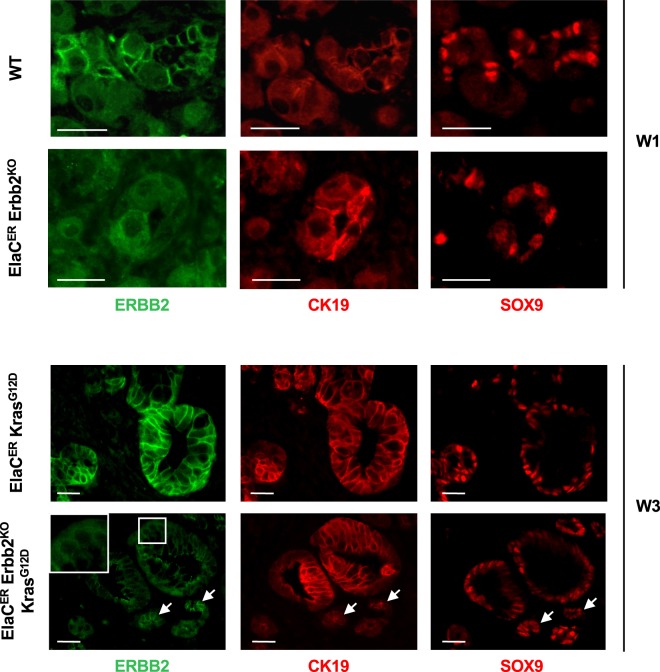
*Erbb2* deletion is efficient and does not affect the expression of metaplastic markers. Immunofluorescent labeling of ERBB2, CK19 and SOX9 in WT and ElaC^ER^ Erbb2^KO^ mice treated 1 week with cerulein (W1), and in ElaC^ER^ Kras^G12D^ and ElaC^ER^ Kras^G12D^ Erbb2^KO^ mice treated 3 weeks with cerulein (W3). Efficient deletion of ERBB2 is detected in ElaC^ER^ Erbb2^KO^ and ElaC^ER^ Kras^G12D^ Erbb2^KO^ mice. Inset in the ElaC^ER^ Kras^G12D^ Erbb2^KO^ panel shows that the ERBB2 labeling is cytoplasmic and non-specific. Expression of the metaplastic markers CK19 and SOX9 is observed in ductal cells (white arrows) which also express ERBB2 as well as in duct-like cells and PanIN. Scale bars = 50 μm.

Next, to investigate the impact of *Erbb2* deletion on neoplastic progression, we generated mice bearing a conditional deletion of *Erbb2* in acinar cells (ElaC^ER^ Erbb2^KO^), combined or not with the expression of Kras^G12D^ (ElaC^ER^ Erbb2^KO^ Kras^G12D^). We analyzed WT, ElaC^ER^ Erbb2^KO^, ElaC^ER^ Kras^G12D^, and ElaC^ER^ Erbb2^KO^ Kras^G12D^ mice treated with cerulein in an acute (W1) or chronic (W3 and W9) setup. Histologically, ADM and mild inflammation were observed in all mice, either during acute treatment (W1) or chronic treatment (W3 and W9) (Fig. 2A). PanINs were also detected in ElaC^ER^ Kras^G12D^ and ElaC^ER^ Erbb2^KO^ Kras^G12D^ mice during acute and chronic treatments, and no difference in their grade or frequency was observed between these two genotypes (Fig. 2A). Alcian Blue staining confirmed this result: no Alcian Blue-positive lesions were observed in WT and ElaC^ER^ Erbb2^KO^ mice (data not shown) whereas Alcian-Blue-positive lesions were found in equal numbers in ElaC^ER^ Kras^G12D^ and ElaC^ER^ Erbb2^KO^ Kras^G12D^ mice (Fig. 2B). These numbers increased with the duration of cerulein treatment (Fig. 2C–D). We verified that *Erbb2* was deleted in ElaC^ER^ Erbb2^KO^ and ElaC^ER^ Erbb2^KO^ Kras^G12D^ mice: ERBB2 immunolabeling confirmed the absence of ERBB2 expression in ADM and PanIN in the pancreas after cerulein treatment (Fig. 3 and Supplementary Fig. S2). The deletion was partial since quantification of *Erbb2* deletion indicated a 6-fold reduction in the number of cells expressing ERBB2 in ElaC^ER^ Erbb2^KO^ Kras^G12D^ pancreata compared to ElaC^ER^ Kras^G12D^ pancreata after 9 weeks of cerulein treatment (Supplementary Fig. S3A). In contrast, an identical proportion of cells expressed EGFR in these pancreata (Supplementary Fig. S3B). Additional confirmation of *Erbb2* deletion in the ElaC^ER^ Erbb2^KO^ Kras^G12D^ pancreata was obtained by genetic lineage tracing: addition of a ROSA26R^YFP^ reporter allele in ElaC^ER^ Kras^G12D^ and ElaC^ER^ Erbb2^KO^ Kras^G12D^ mice enabled to show that a subset of cells expressing YFP as a results of ElaC^ER^-induced recombination also displayed lack of detectable ERBB2 (Supplementary Fig. S3C). These results indicated that, at the histological level, *Erbb2* deletion does not impact the presence or frequency of ADM and PanIN lesions.

### ERBB2-positive and ERBB2-deficient PanIN share the same molecular features

To investigate whether ADM and PanIN formed from ERBB2-positive and ERBB2-deficient acinar cells were similar at the molecular level, we first performed immunolabeling for SOX9 and CK19, two ADM and PanIN markers^16^. After induction of acute (W1) or chronic (W3) pancreatitis, SOX9 and CK19 expression was detected in metaplastic acini of WT and ElaC^ER^ Erbb2^KO^ mice (Fig. 3). SOX9 and CK19 were also expressed in PanINs of ElaC^ER^ Kras^G12D^ and ElaC^ER^ Erbb2^KO^ Kras^G12D^ mice treated for 1 or 3 weeks with cerulein (Fig. 3 and Supplementary Fig. S2). Collagen deposition and immune cell infiltration were analyzed: no difference was observed in the absence or presence of ERBB2 (Supplementary Fig. S4).

**Figure 4:**
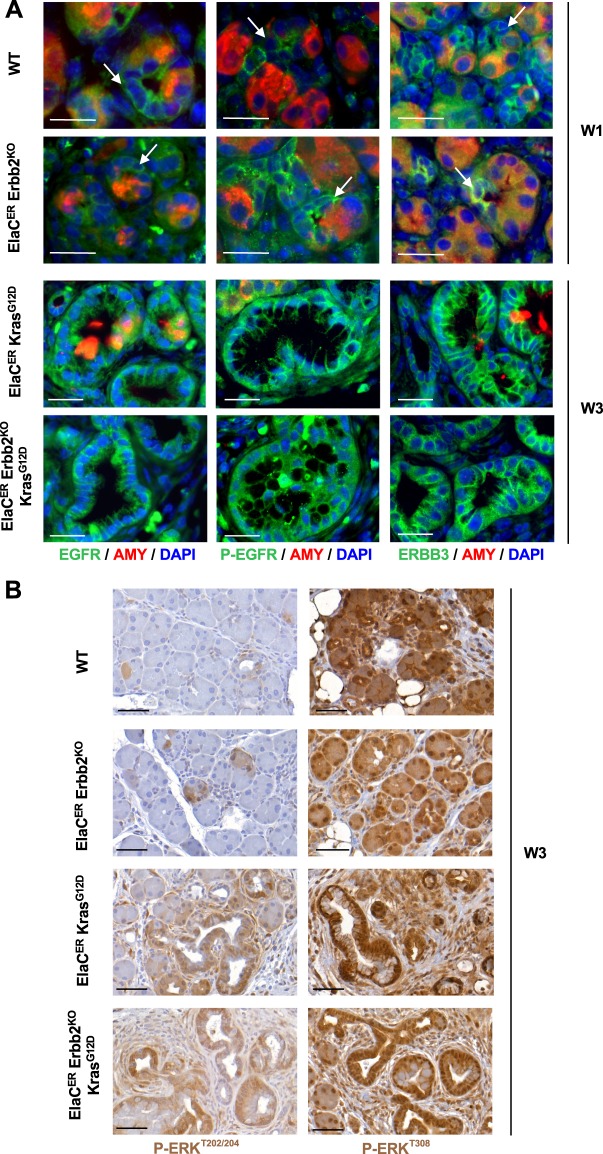
ERBB family members and downstream effectors of ERBB signaling are not affected by *Erbb2* loss. (**A**) Immunofluorescent labeling for EGFR, P-EGFR, ERBB3, and Amylase in WT and ElaC^ER^ Erbb2^KO^ mice treated for 1 week with cerulein (W1) and in ElaC^ER^ Kras^G12D^ and ElaC^ER^ Erbb2^KO^ Kras^G12D^ mice treated for 3 weeks with cerulein (W3). EGFR and ERBB3 are both expressed, and EGFR is activated in ADM in WT and ElaC^ER^ Erbb2^KO^ pancreas (white arrows) and in PanIN present in ElaC^ER^ Kras^G12D^ and ElaC^ER^ Erbb2^KO^ Kras^G12D^ pancreas. DAPI staining visualizes cell nuclei. Scale bars = 50 μm. (**B**) Immunohistochemical staining for P-ERK^T202/204^ and P-AKT^T308^ after 3 weeks of cerulein treatment (W3). Similar proportions of ADM are positive for P-ERK and P-AKT in WT and ElaC^ER^ Erbb2^KO^ mice whereas a large number of PanIN are stained by both antibodies in ElaC^ER^ Kras^G12D^ and ElaC^ER^ Kras^G12D^ Erbb2^KO^ mice. High activity of the AKT pathway is detected in the pancreata of the different genotypes. Scale bars = 50 μm.

The lack of detectable effect of *Erbb2* deletion on ADM and PanIN formation, prompted us to assess whether increased expression or activity of other ERBB family members would compensate for *Erbb2* loss. Since EGFR and ERBB3 reportedly promote PDAC tumorigenesis^8,9,17^, we performed immunolabeling for EGFR, P-EGFR^Y845^ (as a surrogate of EGFR activity) and ERBB3. Their expression was similar in ADM and PanIN lesions present in the different genotypes, irrespective of the presence or absence of ERBB2 (Fig. 4A, Supplementary Figs. S5 and S6). Thus, we conclude that *Erbb2* deletion does not significantly affect the expression or activity of other ERBB family members. Yet, the presence of the other ERBB proteins might be sufficient to compensate for the loss of ERBB2.

Moreover, the RAS/MAPK and PI3K/AKT pathways are normally activated by ERBB2 and are involved in the formation and progression of pancreatic neoplastic lesions^18–20^. In acute or chronic cerulein treatment (W1, W3 or W9), ERK phosphorylation, a marker of MAPK pathway activity, was observed in a limited number of metaplastic acini of WT mice whereas high levels of ERK phosphorylation were detected in PanIN in ElaC^ER^ Kras^G12D^ pancreas (Fig. 4B and Supplementary Fig. S7). Similar observations were made in the absence of Erbb2 in ElaC^ER^ Erbb2^KO^ and ElaC^ER^ Erbb2^KO^ Kras^G12D^ mice (Fig. 4B and Supplementary Fig. S7). The same conclusions were drawn when analysing the AKT pathway: high activity was observed in the pancreas of the various genotypes using a P-AKT^T308^ antibody (Fig. 4B and Supplementary Fig. S7) or a P-AKT^S473^ antibody (data not shown). This indicates that *Erbb2* loss does not significantly perturb the activity of the RAS/MAPK and PI3K/AKT pathways.

### The differential roles for EGFR and ERBB2 in mouse ADM and PanIN development result from signaling properties of the ERBB pathway

Despite that ERBB2 is the preferred dimerization partner of EGFR, its expression is dispensable for ADM and PanIN formation, in contrast to EGFR. To understand this counter-intuitive observation, we investigated how EGFR and ERBB2 control the dynamics of signaling initiation by building an experiment-based mathematical model.

**Figure 5:**
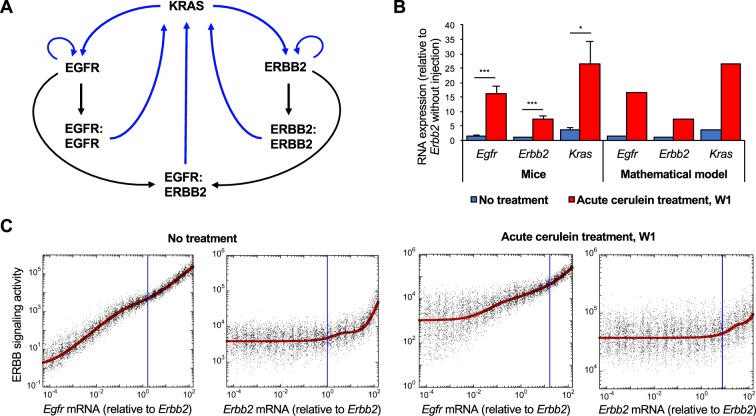
Impact of ERBB2 expression on the activity of ERBB signaling pathway in mice. (**A**) Scheme of the minimal molecular network defining the regulations between KRAS, EGFR, and ERBB2. (**B**) Relative RNA expression levels of mouse *Egfr*, *Erbb2* and *Kras* in the absence (blue bars) or in the presence of acute cerulein treatment (red bars), as determined experimentally in mice (left) and predicted by mathematical modeling (right). Expression data are mean +/− SD, n ≥ 3. *, p < 0.05 and *** p < 0.001. (**C**) Modeling ERBB signaling activity (red curves) as a function of *Egfr* or *Erbb2* mRNA in the absence (left panels) or in the presence of acute cerulein treatment (right panels). Each black dot represents a cell in a heterogeneous cell population where 50% of uniform random variations are considered around the basal value of each parameter (see Supplementary Information for details). Vertical blue lines correspond to the measured mean expression levels of *Egfr* and *Erbb2* with or without inflammation as shown in (**B**). These expression levels are relative to *Erbb2* expression in the absence of treatment.

This model represents a regulatory network composed of interacting KRAS, EGFR and ERBB2 (Fig. 5A). The interactions consist of direct or indirect functional links, namely protein-protein and epistatic relationships that have been experimentally validated^6,8,9,12,21–25^. Indeed, EGFR and ERBB2 monomers can reversibly form EGFR:EGFR homodimers, ERBB2:ERBB2 homodimers or EGFR:ERBB2 heterodimers. These dimers participate in the transcriptional regulation of KRAS, EGFR and ERBB2, and can activate KRAS. KRAS can stimulate transcription of *EGFR* and *ERBB2*^6,8,9,16,21,22,24,26^. The mathematical model consists of a set of kinetic equations describing each interaction in the network, namely the temporal evolution of the expression levels of each network component. It includes mRNA and protein forms of EGFR, ERBB2 and KRAS, and considers inactive (GDP-bound) and active (GTP-bound) KRAS, as well as the monomers, homodimers and heterodimers of EGFR and ERBB2. In the model, ERBB signaling activity is then defined by the sum of the protein expression levels of EGFR and ERBB2 homodimers, EGFR:ERBB2 heterodimers, and active forms of KRAS that are present in a given condition (Supplementary Information). The model’s quantitative assumptions, the equations and the parameter values used in the simulations are described in Supplementary Information.

First, we calibrated the mathematical model based on the expression levels of *Egfr*, *Erbb2* and *Kras* mRNAs measured in the pancreas of WT mice, in the absence or in the presence of acute cerulein treatment (W1), which induces ADM and increased expression of *Egfr*, *Erbb2* and *Kras* mRNAs. Protein expression levels were estimated from literature data (Supplementary Table 4 and ref. ^27^). When introducing in the model the measured *Erbb2* expression levels in normal and cerulein-treated condition, the model faithfully predicted the corresponding expression of *Egfr* and *Kras* (Fig. 5B). The model was then used to simulate the impact of variations of *Egfr* and *Erbb2* mRNA expression on the dynamics of ERBB signaling defined as above. Our model predicted that with or without cerulein treatment, a decrease in *Egfr* mRNA, starting from the control value (blue vertical line in Fig. 5C) strongly affects ERBB signaling activity, while a decrease of *Erbb2* mRNA does not (Fig. 5C, red curves). This prediction is robust since the predicted effects of *Egfr* and *Erbb2* mRNAs on ERBB signaling are maintained when we model a heterogeneous cell population by introducing 50% of uniform random variations around the basal value of each parameter (Fig. 5C: each black dot corresponds to the simulation for one cell in the heterogeneous population). We concluded that a quantitative mathematical model of the interactions between KRAS, EGFR and ERBB2, and calibrated on mouse expression data, suggests that EGFR is critical for ERBB signaling activity in the studied context, while ERBB2 is dispensable. This is in line with their roles in ADM and PanIN development as shown in the present work for ERBB2 and by others for EGFR^8^.

### Modeling ERBB signaling suggests that ERBB2 is dispensable for development of human PDAC

**Figure 6:**
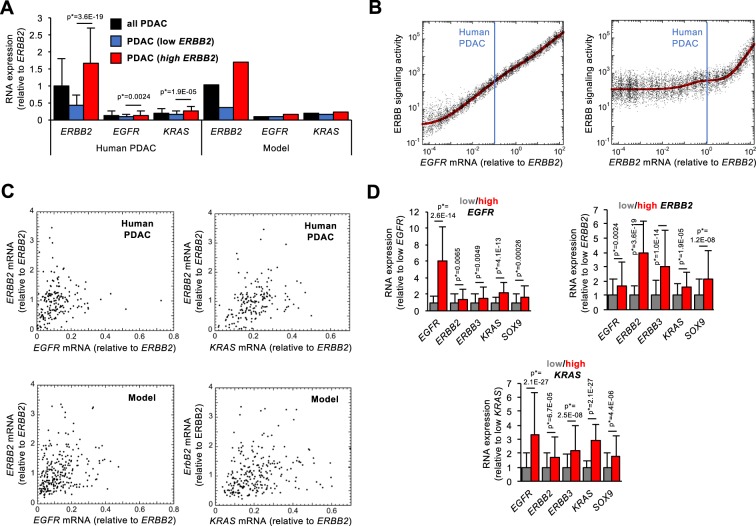
Impact of *EGFR* and *ERBB2* expression on ERBB signaling activity in human PDAC. (**A**) Left: RNA expression of *EGFR*, *ERBB2* and *KRAS* in all PDAC from TCGA (black bars, n = 178), in tumors with lowest (n = 50, blue bars) or highest (n = 50, red bars) *ERBB2* expression. Right: Calibration of the mathematical model on the RNA expression levels available in TCGA. mRNA expression levels were normalized to the mean expression level of *ERBB2* mRNA in all PDAC. (**B**) Modeling the predicted impact of *EGFR* and *ERBB2* expression levels on ERBB signaling activity in human PDAC (red curves). Each black dot is a PDAC patient of a heterogeneous population where 50% of uniform random variations are considered around the basal value of each parameter. Vertical blue lines correspond to the expression levels of *EGFR* and *ERBB2* (relative to the mean ERBB2 expression in all tumors). For (**A,B**), parameter values are described in Supplementary Information. (**C**) Expression levels of *ERBB2* as a function of *EGFR* (left) or *KRAS* (right) in all human PDAC (upper panels, n = 178) and in the mathematical model for a heterogeneous population of 250 PDAC patients with 50% of random uniform variations of parameter values (bottom panels). (**D**) RNA expression levels in tumors with lowest (n = 50, grey bars) or highest (n = 50, red bars) expression of *EGFR* (left), *ERBB2* (middle) or *KRAS* (right). Data are means +/− SD. p* values were adjusted with Benjamini-Hochberg corrections considering the entire transcriptome.

Taking advantage of the robustness of the model and of its ability to faithfully predict ERBB signaling in mice, we next applied it to human pancreas. The structure of the EGFR/ERBB2/KRAS network (Fig. 5A) had been experimentally validated in mice. In humans, we validated the network by verifying whether expression of *EGFR*, *ERBB2* and *KRAS* mRNA was positively correlated in human PDAC (Fig. 6D). The PDAC cohort (n = 178) of TCGA was used as a source of data. By comparing the 50 PDAC samples from the cohort that show the highest expression of either *EGFR*, *ERBB2* or *KRAS*, with the 50 samples that have the lowest expression of the same component, we found that high mean expression of one of the three genes correlated with high mean expression of the two others; similarly, low mean expression of one of the three genes correlated with low mean expression of the two others. This correlation was extended to the mRNA levels of *ERBB3* and *SOX9*, two other components of the ERBB pathway (Fig. 6D)^16^. However, we have not considered ERBB3 as a dimerization partner for EGFR and ERBB2 in our mathematical model, since the ERBB3 ligands NRG1 and NRG2 are not, or marginally, expressed in human pancreas (Supplementary Fig. S8A)^28^, in human PDAC (Supplementary Fig. S8B), and in mouse pancreas (Supplementary Fig. S8C). In addition, phosphorylation of ERBB3 was not detected in mouse pancreas (Supplementary Fig. S8D). Together, these results indicate that ERBB3 is not active in the pancreas.

We next recalibrated the mathematical model on the expression levels of human *ERBB2*, *EGFR* and *KRAS* mRNAs detected in three different PDAC conditions (RNASeq data from TCGA): (i) in all tumors (n = 178), (ii) in the 50 tumors with lowest or (iii) highest expression of *ERBB2* mRNA. This recalibration on human data was performed by modifying only the transcription rates of *EGFR*, *ERBB2* and *KRAS* (see Supplementary Information). As in mice, the protein expression levels in human conditions were estimated from literature data (Supplementary Information). By introducing in the model the mean *ERBB2* expression values measured in all tumors, the model faithfully predicted the measured expression of *EGFR* and *KRAS* (Fig. 6A, black bars). Similarly, when introducing in the model the mean *ERBB2* values measured in the 50 PDAC samples that had the lowest *ERBB2* expression (Fig. 6A, blue bars) or the *ERBB2* values measured in the 50 samples with the highest *ERBB2* expression (Fig. 6A, red bars), the model faithfully predicted the corresponding expression of *EGFR* and *KRAS*. This result showed that the model was properly calibrated with human data to enable simulation of *EGFR* or *ERBB2* variations.

We then simulated the steady-state levels of ERBB signaling activity, defined as above, as a function of the concentration of *EGFR* and *ERBB2* mRNAs (Fig. 6B, red curves). The model showed, like in mice, that reduction in *EGFR* starting at the control value (blue vertical line in Fig. 6B) reduces ERBB signaling while reduced *ERBB2* expression does not affect ERBB signaling. As in mice, in a heterogeneous patient PDAC population, the model predicted a robust network dynamics even in the presence of 50% of random fluctuations of the parameter values (Fig. 6B, black dots).

In addition to having robust network dynamics towards random fluctuations in parameter values (Fig. 6B), the model exhibits, with 50% of random fluctuation on parameter values, a level of heterogeneity in *EGFR*, *ERBB2* and *KRAS* mRNA expression similar to that in the human PDAC (Fig. 6C). This indicates that fluctuations in the network dynamics are similar in the model and in the human PDAC condition.

In conclusion, our analysis suggests that in human pancreas at PDAC stage, like in mouse pancreas in precancerous lesions, EGFR, but not ERBB2, is critical for ERBB signaling.

### Transcription and translation rates of EGFR and ERBB2 are critical for ERBB signaling

*In silico* modeling, based on human PDAC condition, enables to determine why ERBB2 is dispensable for ERBB signaling by analysing the parameters that are critical in the network. To address this question, we plotted the steady-state levels of ERBB signaling activity as a function of *ERBB2* (Supplementary Fig. S9) or *EGFR* mRNA levels (Supplementary Fig. S10) in the presence of a ten-fold increase or decrease of each parameter value. These analyses predicted that reducing *ERBB2* mRNA has no impact on signaling except if the transcription (*T*_EGFR_) and translation (*V*_SEGFR_) rates of EGFR are very low (Supplementary Fig. S9A,C). All other parameters characterizing ERBB2, EGFR and KRAS expression, degradation, dimerization and activity have little or no influence on the effect of *ERBB2* mRNA levels on ERBB signaling (Supplementary Fig. S9B,D–Q). In parallel, simulating a decrease in *EGFR* mRNA levels strongly reduces ERBB signaling except if the transcription (*T*_ERBB2_) and translation (*V*_SERBB2_) rates of ERBB2 are very high, or if the homodimerization rate (*k*_ASS2_) of ERBB2 strongly increases (Supplementary Fig. S10A,D,G). Other parameter values do not affect the function of EGFR. In other terms, expression of ERBB2 would only compensate for low EGFR levels when ERBB2 expression or homodimerization is high.

Finally, as observed experimentally^8^, a rise in the expression level of *EGFR* is predicted to increase the activity of ERBB signaling. Rising *ERBB2* expression is also predicted to stimulate ERBB signaling, but only at expression levels that are higher than physiological levels, i.e. when ERBB2 is overexpressed. These predictions fit with published experimental data showing how ERBB2 and EGFR promote development of precancerous lesions^12,24,25^.

Thus, the model suggests that ERBB signaling activity is robustly dependent on EGFR but independent of ERBB2, except if ERBB2 is strongly overexpressed.

### ERBB2 overexpression stimulates ERBB signaling activity

**Figure 7:**
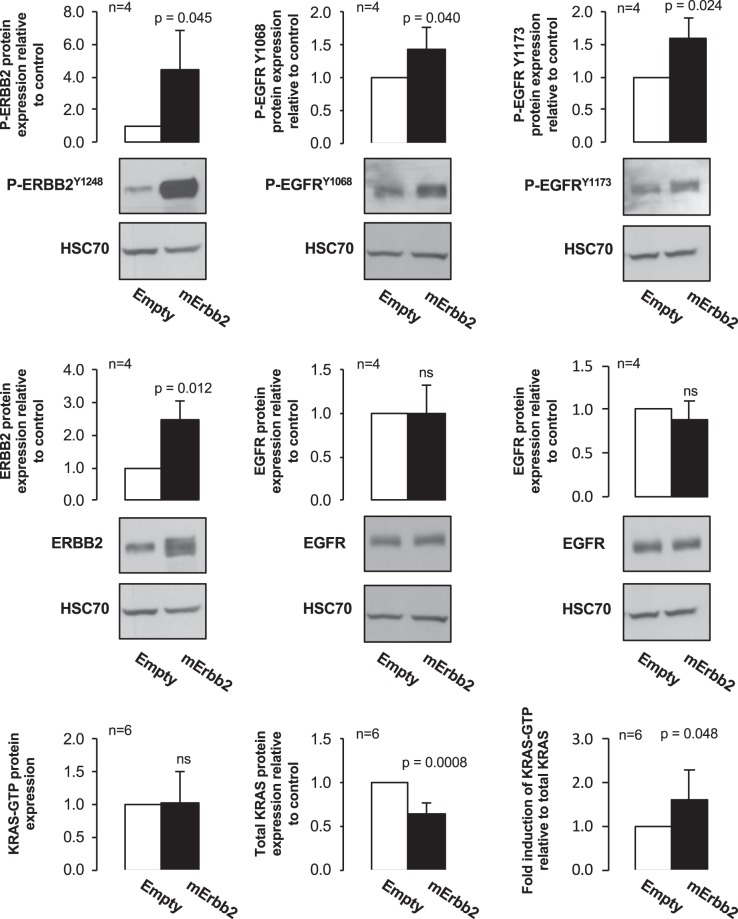
ERBB2 overexpression partially impacts ERBB signaling activity. Representative immunoblots of proteins extracted from PANC1 cells infected with empty or mErbb2 lentiviruses. Levels of ERBB2, EGFR and their phosphorylated forms, as well as the KRAS-GTP/KRAS ratio, were quantified; they increased significantly after mErbb2 lentiviral infection. Fold inductions are mentioned as mean +/− SD; ns, p > 0.05. Phosphorylated and non-phosphorylated forms of ERBB2 and EGFR were detected on the same blots. Consequently, HSC70 loading controls were the same. Blots were cropped and full-lengt blots are avaible in Supplementary Figs. S13 to S15.

To experimentally validate our mathematical model in the presence of increased expression of ERBB2, PANC1 and MiaPaCa-2 cells were infected with empty or mErbb2 lentiviral vectors (Fig. 7 and Supplementary Fig. S11). ERBB2 overexpression was associated with an increase of P-ERBB2^Y1248^, P-EGFR^Y1068^ and P-EGFR^Y1173^ in PANC1 cells, and an increase of P-ERBB2^Y1248^ and P-EGFR^Y1068^ in MiaPaCa-2 cells. KRAS-GTP/KRAS ratio also increased in PANC1 cells, which supported that ERBB2 signaling was stimulated. We concluded that ERBB2 overexpression leads to an increased ERBB signaling activity in PANC1 cells, as predicted by our mathematical model.

## Discussion

EGFR and ERBB2 are often expressed or overexpressed in human PDAC^29–32^, and their overexpression is frequently correlated^33^. In human PDAC cell lines, EGFR is known to stimulate proliferation^9^, and in mouse models with oncogenic KRAS^G12D^ expression, deletion of *Egfr* impedes PanIN and PDAC formation^8,9^. A similar mouse model with deletion of *Erbb2* has not been generated. However, overexpression of ERBB2 in mouse acinar cells leads to chronic pancreatic inflammation and increased KRAS expression and activity^12^. In this system, despite the presence of inflammation, PanIN and PDAC are not observed, likely because KRAS is not mutated. Recently, ERBB2, in conjonction with oncogenic KRAS^G12D^, was shown to promote formation of pancreatic neoplastic lesions^13^. However, a mutant form of activated rat ERBB2 was used, and, unlike in humans, its expression started in embryonic pancreatic progenitors which are notoriously overresponsive to oncogenic stress. Also, the selected ERBB2 mutation has to our knowledge not been found in human PDAC. Recently, ERBB2 amplification and gain-of-function mutations have been identified in human PDAC patients, and mutations of ERBB2 and KRAS co-occur. Cell culture studies revealed that inhibition of ERBB2 in PDAC cell lines overexpressing ERBB2 represses proliferation and invasion^34^. Finally, heterogeneous expression of EGFR was detected in a mouse model of PDAC with *Kras*^*G12D*^ and T*p53*^*R172H*^ mutations, whereas ERBB2 expression and ERK activation were elevated and homogeneous in PanIN, PDAC and metastases^6^. Since ERBB2 is the preferred dimerization partner of EGFR^10,11^, these data suggest that ERBB2 plays an important role in PanIN/PDAC formation.

Here, we explored the role of ERBB2 in development of ADM and PanIN and in ERBB signaling. We surprisingly found that deletion of *Erbb2* does not impact ADM and PanIN formation in mice. In agreement with our experimental observations, our mathematical model, which considers dimerization of EGFR and ERBB2 and activation of KRAS, predicts that overexpression of *EGFR* or *ERBB2* promotes ERBB signaling, while downregulation of *EGFR* deletion decreases ERBB signaling. The mathematical model also predicts that downregulation *ERBB2* does not impact ERBB signaling, except if it is overexpressed. This prediction fits with the fact that ERBB2 is a tyrosine kinase receptor that only homodimerizes when it is overexpressed, as was shown in breast cancer^35^. In other terms, its oncogenic potential is only achieved when overexpressed, in contrast to EGFR. Our *in vitro* experiments using PANC1 cells are in agreement with our mathematical model. We acknowledge that in these experiments the increased activity of ERBB signaling is modest following ERBB2 overexpression. However, this overexpression is obtained by lentiviral infection and only reaches 2 to 2.5-fold; in our model, this corresponds to the onset of ERBB2-induced stimulation of ERBB signaling, suggesting that larger overexpression will lead to an even higher stimulation of signaling.

Transcript levels of *EGFR*, *ERBB2* and *KRAS* are heterogeneous in the human PDAC samples from TCGA (Fig. 6C). The mathematical model for a heterogeneous cell population indicates that 50% of random variation around the basal value of each parameter reproduces tumor heterogeneity. It further indicates that stochastic gene expression in ERBB signaling may be a source of heterogeneity in PDAC and, as a consequence, could cause heterogeneous response to therapy.

The core network composed of EGFR, ERBB2 and KRAS described here, is regulated by other genes. SOX9 activates the ERBB pathway, resulting in PDAC initiation^16^, and KRAS can activate a SRC/PEAK1/ERBB2 amplification loop in PDAC^36^. The impact of the core network regulators is taken into account in the model, namely in the parameters that determine the synthesis and degradation rates of EGFR, ERBB2 and KRAS.

In conclusion, our data show that deletion of *Erbb2* in acinar cells does not affect ERBB signaling activation during ADM and/or PanIN development, unlike deletion of *Egfr*. In contrast, increased expression of ERBB2 stimulates ERBB signaling. Therefore, EGFR and ERBB2 differentially impact ERBB signaling during pancreatic cancer tumorigenesis, highlighting the need to consider patient’s specific characteristics of ERBB signaling to optimize therapeutic treatment.

## Materials and methods

### Mouse experimentation

*Elastase-Cre*^*ERT2*^ (ElaC^ER^) and *LsL-Kras*^*G12D*^ (here after called Kras^G12D^) mice have been described^37,38^. *Erbb2*^*flox*^ mice were obtained from Carmen Birchmeier^39^, and were crossed with PGK-Cre mice to obtained *Erbb2*^*+/−*^ mice. The latter were crossed with *Erbb2*^*flox/flox*^ to obtain *Erbb2*^*flox/−*^ mice (hereafter called Erbb2^KO^). All strains were maintained in a CD1-enriched background. Adult mice (6-week old) were treated with 30 mg/ml of tamoxifen (Sigma, Overijse, Belgium) and 0.3 mg/ml of 4-hydroxytamoxifen (Sigma) dissolved in corn oil (Sigma). Animals received humane care according to the Directive 2010/63/EU of the European Parliament and of the Council on the protection of animals used for scientific purposes; the Belgian law of May 29, 2013 on protection of animals used for experimentation, updated on September 7, 2017 by the Brussels Region. Protocols were approved by the Animal Welfare Committee of the Université catholique de Louvain with license number 2017/UCL/MD/020. A detailed description of cerulein treatments is provided in Supplementary Information.

### Immunohistochemistry and immunofluorescence

Pancreata were fixed for 4 or 6 hours in 4% paraformaldehyde at 4 °C and then embedded in paraffin. Antigen retrieval was performed in a microwave oven by incubating 6 µm tissue sections in 10 mM citrate buffer (pH 6) for 10 minutes (or in PreTreatment Module, Lab Vision, for 2 hours). Sections were then permeabilized in PBS/0.3% Triton X-100 (Sigma) buffer for 5 minutes and blocked with PBS/3% low-fat milk/10% bovine serum/0.3% Triton X-100 for 45 minutes at room temperature.

Primary antibodies were diluted in blocking buffer and incubated overnight at 4 °C (See Supplementary Table 1). Secondary antibodies and streptavidin-POD conjugate (1/1000) were diluted in PBS/10% bovine serum/0.3% Triton X-100 for 1 hour at 37 °C. Pictures were taken using a Cell Observer Spinning Disk confocal microscope (Zeiss, Zaventem, Belgium) after immunofluorescence labelling, or with a Mirax imaging system (Zeiss) after immunohistochemical staining.

### Cell culture experiments

PANC1 and MiaPaCa-2 cell lines were grown in DMEM (Lonza, Leusden, Netherlands) supplemented with fetal bovine serum (FBS) 10% (Merck, Darmstadt Germany), sodium pyruvate (1 mM) (Gibco™, Waltham, MA, USA), penicillin-streptomycin 1% (Gibco™) and amphotericin B 1% (Gibco™). For MiaPaCa-2 cells, horse serum 2.5% (Gibco™) was added to the medium. HEK-293T cells were grown in DMEM (Lonza) supplemented with FBS 10% (Merck), penicillin-streptomycin 1% (Gibco™) and amphotericin B 1% (Gibco™).

Lentiviral particles were obtained by calcium phosphate-mediated transfection of HEK-293T. HEK-293T cells were seeded and transfected with plasmids encoding proteins involved in viral packaging (pRSV-REV, pCMV-dR8.2 dvpr and pCMV-VSV-G) as well as pLenti-PGK-Empty or pLenti-PGK-*mErbb2*. These plasmids and their constructions are detailed in Supplementary Information. Lentiviruses were collected, filtered and concentrated with Lenti-X™ Concentrator (Clontech, Mountain View, CA, USA). Concentrated lentiviruses were added to target cells (PANC1) that were selected with hygromycin B (Sigma) at 400 µg/ml (MiaPaCa-2) or 600 µg/ml (PANC1) for 2 weeks. For Western Blot experiments and KRAS pull-down assay, PANC1 cell lines were collected 48 hours after plating. Detailed experimental protocols about Western Blot and KRAS pull-down assay can be found in Supplementary Information.

### RNA extraction and analysis

Total RNA was isolated from fragments of pancreas using Trizol (Invitrogen, Life technologies). cDNA was synthesized using MMLV reverse transcriptase (Invitrogen, Life technologies) according to manufacturer’s protocol. Gene expression was quantified by RT-qPCR using Kapa SYBR Fast 2X Universal Master Mix (Sopachem). mRNA levels were normalized with the geometric mean between *Gapdh* and *18S*. *Gapdh* Fwd: GGTCCTCAGTGTAGCCCAAG, *Gapdh* Rev: AATGTGTCCGTCGTGGATCT; 18S Fwd: GTAACCCGTTGAACCCCATT, 18S Rev: CCATCCAATCGGTAGTAGCG; *Egfr* Fwd: GCCATCTGGGCCAAAGATACC, *Egfr* Rev: GTCTTCGCATGAATAGGCCAA; *Erbb2* Fwd: TAACTGGACCCCAGCCTATG, *Erbb2* Rev: AACGGAGAATGACCCTGTTG; *Kras* Fwd: ACAGGCTCAGGAGTTAGCAAGGA, *Kras* Rev: AAGGCATCGTCAACACCCTGTC. 2^−ΔCt^ method was used for gene quantification in all figures. Then, the expression of each gene was further normalized to the expression of *Erbb2*.

### RNASeq data and statistical analysis

RNASeq data were from the PDAC cohort in the The Cancer Genome Atlas (TCGA) database (http://firebrowse.org/). We normalized the mRNA expression levels to the expression of *ERBB2* mRNA. Data are means ± SD. Significance was assessed by Student t-test or by Student t-test with Benjamini-Hochberg corrections considering the entire transcriptome (Fig. 6).

### Mathematical model

The mathematical model is defined by a set of kinetic equations describing the temporal evolution of the mRNA and protein expression levels of EGFR, ERBB2 and KRAS. The mathematical model with the kinetic equations and the parameter values are described in Supplementary Information. Numerical simulations were performed with XPPAUTO (http://www.math.pitt.edu/~bard/xpp/xpp.html) and Matlab.

## Supplementary information

Supplementary Information.
