# Supplementary material for: Differential impact of the ERBB receptors EGFR and ERBB2 on the initiation of precursor lesions of pancreatic ductal adenocarcinoma
Source: Sci Rep. 2020 Mar 23;10:5241. doi: 10.1038/s41598-020-62106-8 (PMC7090067; doi:10.1038/s41598-020-62106-8)
Supplement: Supplementary file 1 — Supplementary Information. [file 41598_2020_62106_MOESM1_ESM.docx]

**Differential impact of the ERBB receptors EGFR and ERBB2 on the initiation of precursor lesions of pancreatic ductal adenocarcinoma**

**Nora Meyers*, Claude Gérard*, Frédéric P. Lemaigre^#^, Patrick Jacquemin^#^**

Université catholique de Louvain, de Duve Institute, Brussels, Belgium

* NM and CG contributed equally to the present work

^#^ FPL and PJ share senior authorship

**Supplementary Information**

1. Mouse experimentation: cerulein treatment and experimental quantifications 3

2. Plasmids and sub-cloning procedure 3

3. Western Blotting 4

4. KRAS pull-down assay 5

5. Description and calibration of the mathematical model 5

6. Structure of the network and parameter conditions 6

7. Supplementary Tables 9

8. Supplementary Figure Legends 15

9. Supplementary Figures 22

10. Supplementary references 40

1. **Mouse experimentation: cerulein treatment and experimental quantifications**

Seven days after tamoxifen treatment, mice received acute or chronic treatment of cerulein (Sigma) to induce pancreatitis. The initial day of treatment was day 1 (D1). Acute pancreatitis was induced by 7 hourly intraperitoneal injections of cerulein (125 μg/kg), every other day, for 5 days. To induce chronic pancreatitis, at the end of the acute treatment, the mice continued to receive a daily injection of cerulein, 5 days per week, for 2 or 8 weeks. Mice were sacrificed at the beginning of the second week for the acute treatment (W1), or at the beginning of the fourth or tenth week for the chronic treatments of 3 and 9 weeks (W3 or W9), respectively (Fig. 1A). 3 to 7 mice of each genotype were used per stage.

Quantification of Alcian Blue-positive and tissue areas (at least two sections of pancreas of three mice for each genotype) in ElaC^ER^ Kras^G12D^ and ElaC^ER^ Erbb2^KO^ Kras^G12D^ mice treated for 1, 3 and 9 weeks with cerulein (W1, W3 and W9, respectively) was performed using the CaseViewer software (3DHistech, Budapest, Hungary).

To determine the percentage of cells positive for EGFR and ERBB2 in PanIN lesions of ElaC^ER^ Kras^G12D^ and ElaC^ER^ Erbb2^KO^ Kras^G12D^ mice, around 600 cells per genotype were counted.

1. **Plasmids and sub-cloning procedure**

*Erbb2* murine sequence was amplified with the primers 5’ TTCCTGTACACCGGAGCCGCAGTGATCATCATG 3’ and 5’ GGAATGTACAAGGATGTCTGCACATGTGACCTCA 3’ and sub-cloned in the pLenti-PKG vector. PCR was performed using KAPA HiFi HotStart PCR Kit (Takara, Mountain View, CA, USA) according to the manufacturer’s protocol. The amplification product was digested with BsrGI (New England Biolabs, Ipswich, MA, USA) for 1 hour at 37°C, as well as pLenti-PGK-KRAS4B(G12V) (Addgene #35633) in order to remove the KRAS4B(G12V) sequence and generate BsrGI restriction enzyme sites in the pLenti-PKG vector. T4 DNA ligase (Thermo Fisher Scientific, Waltham, MA, USA) was used to perform ligation reactions for 150 minutes at 22°C, with the digested plasmid in the presence or the absence of the digested *mErbb2* amplification product, to create pLenti-PGK-*mErbb2* and pLenti-PGK-Empty, respectively.

# **Western Blotting**

Proteins were extracted with RIPA buffer (50mM Tris-HCl pH7.4, 150mM NaCl, NP-40 1%, sodium deoxycholate 0.25%, 1mM sodium orthovanadate) complemented with protease and phosphatase inhibitors (Roche, Basel, Switzerland). Cell lysates were sonicated for 15 seconds and centrifuged. Proteins were then dosed using Bradford protein assay. 60 µg of proteins were loaded on SDS-PAGE in polyacrylamide gels 7.5-12% and transferred onto a PVDF membrane (Merck). After blocking, membranes were incubated overnight at 4°C with primary antibody (Supplementary Table 6) in Tris-Buffered Saline (TBS) with Tween-20 0.1% (Sigma) and BSA 5% or milk 5%. Then, membranes were incubated with anti-rabbit (1:2000, Enzo Life Sciences, Plymouth Meeting, PA, USA) or anti-mouse (1:1000, Cell Signaling Technology, Danvers, Massachusetts, USA) secondary antibodies, and proteins were detected using chemiluminescence (Thermo Fisher Scientific) and X-ray film (Thermo Fisher Scientific).

# **KRAS pull-down assay**

KRAS-GTP pull-down was achieved with the K-Ras Activation Assay Kit (Abcam, Cambridge, United Kingdom) according to the manufacturer’s protocol using 1 mg of protein extract. Western blotting was performed to detect Kras-GTP and total KRAS (KRAS-GTP + KRAS-GDP), called KRAS in the corresponding experiment). For KRAS detection, 25 µg of protein extract were used.

1. **Description and calibration of the mathematical model**

The mathematical model was defined by explicit dynamic variables (Supplementary Tables 2 and 3) such as the free form of each mRNA component, i.e. *EGFR* (mEGFR) in Eq. [1], *ERBB2* (mERBB2) in Eq. [2], and KRas (mKRAS) in Eq. [8]. Protein forms for each component were also considered: the monomer of EGFR in Eq. [3] and of ERBB2 in Eq. [5], the homodimer of EGFR (cEGFR) in Eq. [4] and of ERBB2 (cERBB2) in Eq. [6], the heterodimer between EGFR and ERBB2 (cERBB12) in Eq. [7], as well as the inactive form of KRAS (KRAS) in Eq. [9] and its active form (KRAS_A_) in Eq. [10]. All reaction steps in the model rely on mass-action kinetics, except for the Michaelis expressions defining the activation of transcription by EGFR, ERBB2 and KRAS in Eqs. [1], [2], [8] and the activation of KRAS by EGFR and ERBB2 in Eqs. [9] and [10].

For mouse data (Fig. 5), the mathematical model was calibrated on the expression levels, measured by RT-qPCR (expression relative to the geometric mean between *ACTB* and *18S* rRNA), of *EGFR*, *ERBB2* and *KRAS* mRNA. These expression levels were measured in the absence or in the presence of an acute cerulein treatment.

For human data, the mathematical model was calibrated on the expression levels, determined by RNASeq, of *EGFR*, *ERBB2* and *KRAS* mRNA in the PDAC cohort (n = 178) from the The Cancer Genome Atlas, TCGA (see <http://firebrowse.org/)>. To facilitate the comparison between mouse and human data, each component was normalized, in each condition, to the expression levels of ERBB2.

In the model, the total forms of each protein can be calculated as follows:

| ${EGFR}_{TOT}=EGFR+cEGFR+cERBB12$  ${ERBB2}_{TOT}=ERBB2+cERBB2+cERBB12$  ${KRAS}_{TOT}=KRAS+{KRAS}_{A}$  The total activity level of ERBB signaling was calculated as follows:  $SIGNALING=cEGFR+cERBB2+cERBB12+{KRAS}_{A}$ |
| --- |

Note that the steady-state expression level of each component is given by the balance between its rate of synthesis and its rate of degradation. Half-life of mRNA and protein for each component was based on the literature (Supplementary Table 4).

1. **Structure of the network and parameter conditions**

In the model, we considered that EGFR and ERBB2 monomers can reversibly form EGFR:EGFR homodimers, with an association rate *k*_ASS1_, ERBB2:ERBB2 homodimers, with an association rate *k*_ASS2_, or EGFR:ERBB2 heterodimer, with an association rate *k*_ASS3_. These three dimers can activate KRAS, EGFR and ERBB2 transcription [1, 2] and stimulate KRAS protein activity [3]. In turn, the active form of KRAS protein can activate transcription of EGFR and ERBB2 (Fig. 5A, kinetic equations in Supplementary Table 3 and Refs. [1, 4-10]). EGFR:EGFR, ERBB2:ERBB2 and EGFR:ERBB2 can promote the transcription of ERBB2 and EGFR [10, 11]. Finally, these dimers can be degraded with a degradation rate constant *k*_DCEGFR_, *k*_DCERBB2_, *k*_DCERBB12_, respectively, which represent the internalization rates of each dimeric receptor.

We constrained the model’s parameter values with different biological considerations such as the half-life durations of *EGFR*, *ERBB2* and *KRAS* mRNAs and proteins (see Supplementary Table 4). In addition, in physiological conditions, it is known that the formation rate of ERBB2:ERBB2 homodimer is low as a result of electrostatic repulsion [12]. As a consequence, we considered that *k*_ASS2_ is very small, as compared to *k*_ASS1_ and *k*_ASS3_.

In Figure 6A, the basal set of parameter values (Supplementary Table 5) enables to reproduce the mRNA expression levels of *ERBB2*, *KRAS* and *EGFR* in all PDAC samples (black bars). To reproduce the expression levels of these components in PDAC samples with low *ERBB2* mRNA levels (blue bars), the rate of synthesis of *ERBB2*, *TERBB2*, was reduced from 1 to 0.45 while *k*_ARAS_ was changed from 2 to 0.1. To reproduce the condition in PDAC samples with high *ERBB2* mRNA expression levels (red bars), *TERBB2*, was increased from 1 to 1.2 while *k*_ARAS_ was changed from 2 to 10.

1. **Supplementary Tables**

**Supplementary Table 1.** List of primary antibodies used for immunostaining experiments

| Antibodies | Species | Dilution | Reference |
| --- | --- | --- | --- |
| ERBB2 | Rabbit | 1/300 | Dako (#A0485) |
| CK19 | Rat | 1/50 | Developmental Studies Hybridoma Bank (Troma III) |
| SOX9 | Goat | 1/300 | R&D Systems Europe Ltd (#AF3075) |
| Amylase | Mouse IgG2a | 1/500 | Santa Cruz (#SC46657) |
| EGFR | Rabbit | 1/50 | Cell Signaling (#4267) |
| P-EGFR^Y845^ | Rabbit | 1/100 | Invitrogen (#44784G) |
| ERBB3 | Rabbit | 1/200 | Cell Signaling (#12708) |
| p-ERK^T202/204^ | Rabbit | 1/200 | Cell Signaling (#43705) |
| p-AKT^T308^ | Rabbit | 1/100 | Cell Signaling (#2965) |
| GFP | Goat | 1/250 | Abcam (#AB6673) |

**Supplementary Table 2.** Variables of the mathematical model

| **Equations** | **Symbol** | **Definition** |
| --- | --- | --- |
| 1 | *mEGFR* | *EGFR* messenger RNA |
| 2 | *mERBB2* | *ERBB2* messenger RNA |
| 3 | *EGFR* | Monomer of EGFR protein |
| 4 | *cEGFR* | EGFR homodimer |
| 5 | *ERBB2* | Monomer of ERBB2 protein |
| 6 | *cERBB2* | ERBB2 homodimer |
| 7 | *cERBB12* | EGFR : ERBB2 heterodimer |
| 8 | *mRAS* | *KRAS* messenger RNA |
| 9 | *KRAS* | Inactive form of KRAS protein |
| 10 | *KRAS_A_* | Active form of KRAS protein |

**Supplementary Table 3.** Kinetic equations of the mathematical model

$\frac{dmEGFR}{dt}=T_{EGFR}\cdot\left( V_{SM1EGFR}+V_{SM2EGFR}\cdot\left( \frac{cEGFR+cERBB2+cERBB12}{K_{A1}+\left( cEGFR+cERBB2+cERBB12 \right)} \right)+V_{SM3EGFR}\cdot\left( \frac{{KRAS}_{A}}{K_{A2}+{KRAS}_{A}} \right) \right)-k_{DMEGFR}\cdot mEGFR$ [1]

$\frac{dmERBB2}{dt}=T_{ERBB2}\cdot\left( V_{SM1ERBB2}+V_{SM2ERBB2}\cdot\left( \frac{cEGFR+cERBB2+cERBB12}{K_{A1}+\left( cEGFR+cERBB2+cERBB12 \right)} \right)+V_{SM3ERBB2}\cdot\left( \frac{{KRAS}_{A}}{K_{A2}+{KRAS}_{A}} \right) \right)-k_{DMERBB2}\cdot mERBB2$ [2]

$\frac{dEGFR}{dt}=V_{SEGFR}\cdot mEGFR -k_{ASS1}\cdot EGFR\cdot EGFR+k_{D}\cdot cEGFR-k_{ASS3}\cdot EGFR\cdot ERBB2+k_{D}\cdot cERBB12-k_{DEGFR}\cdot EGFR$ [3]

$\frac{dcEGFR}{dt}=k_{ASS1}\cdot EGFR\cdot EGFR-k_{D}\cdot cEGFR-k_{DCERBB1}\cdot cEGFR$ [4]

$\frac{dERBB2}{dt}=V_{SERBB2}\cdot mERBB2 -k_{ASS2}\cdot ERBB2\cdot ERBB2+k_{D}\cdot cERBB2-k_{ASS3}\cdot EGFR\cdot ERBB2+k_{D}\cdot cERBB12-k_{DERBB2}\cdot ERBB2$ [5]

$\frac{dcERBB2}{dt}=k_{ASS2}\cdot ERBB2\cdot ERBB2-k_{D}\cdot cERBB2-k_{DCERBB2}\cdot cERBB2$ [6]

$\frac{dcERBB12}{dt}=k_{ASS3}\cdot EGFR\cdot ERBB2-k_{D}\cdot cERBB12-k_{DCERBB12}\cdot cERBB12$ [7]

$\frac{dmRAS}{dt}=T_{RAS}\cdot\left( V_{SM1RAS}+V_{SM2RAS}\cdot\left( \frac{cEGFR+cERBB2+cERBB12}{K_{A4}+\left( cEGFR+cERBB2+cERBB12 \right)} \right) \right)-k_{DMRAS}\cdot mRAS$ [8]

$\frac{dKRAS}{dt}={V_{SRAS}\cdot mRAS- k}_{ARAS}\cdot KRAS\cdot\left( \frac{cEGFR+cERBB2+cERBB12}{K_{A3}+\left( cEGFR+cERBB2+cERBB12 \right)} \right)+k_{IRAS}\cdot{KRAS}_{A}-k_{DKRAS}\cdot KRAS$ [9]

$\frac{d{KRAS}_{A}}{dt}=k_{ARAS}\cdot KRAS\cdot\left( \frac{cEGFR+cERBB2+cERBB12}{K_{A3}+\left( cEGFR+cERBB2+cERBB12 \right)} \right)-k_{IRAS}\cdot{KRAS}_{A}-k_{DKRAS}\cdot{KRAS}_{A}$ [10]

**Supplementary Table 4.** Calibration of the mathematical model

|  | **Experimental values** | | **Values in the model** | | **Ref.** |
| --- | --- | --- | --- | --- | --- |
|  | **mRNA half-life (h)** | **protein half-life (h)** | **mRNA half-life (h)** | **protein half-life (h)** |  |
| **EGFR** | 8.32 | 12.71 | 8.35 | 12.72 | [13] |
| **ERBB2** | 10.59 | 18.52 | 10.5 | 18.73 | [13] |
| **KRAS** | 6.14 | 22.55 | 6.13 | 22.36 | [13] |

Schwanhausser et al. [13] have shown that the mean protein/mRNA ratio measured over 5000 different genes is close to 3000. This ratio can vary extensively among genes over multiple orders of magnitude ranging from 1 to 10^7^. In the mathematical model, the mean protein/mRNA ratio of all network components is equal to 2158, which is very close to the mean protein/mRNA ratio measured experimentally in Schwanhausser et al. [13]. h, hours.

**Supplementary Table 5.** Parameter values of the mathematical model

| **Symbol** | **Definition** | **Numerical value^*^** |
| --- | --- | --- |
| *TEGFR* | Parameter multiplying all transcription rates of EGFR | 1 (human) ; 7.5 (mice) |
| *TERBB2* | Parameter multiplying all transcription rates of ERBB2 | 1 (human) ; 0.6 (mice) |
| *TRAS* | Parameter multiplying all transcription rates of KRAS | 1 (human) ; 10.5 (mice) |
| *V*_SM1EGFR_ | Basal transcription rate of EGFR | 0.0035 |
| *V*_SM2EGFR_ | Transcription rate of EGFR regulated by EGFR and ERBB2 dimers | 0.01 |
| *V*_SM3EGFR_ | Transcription rate of EGFR regulated by the active form of KRAS | 0.006 |
| *V*_SM1ERBB2_ | Basal transcription rate of ERBB2 | 0.04 |
| *V*_SM2ERBB2_ | Transcription rate of ERBB2 regulated by EGFR and ERBB2 dimers | 0.045 |
| *V*_SM3ERBB2_ | Transcription rate of ERBB2 regulated by the active form of KRAS | 0.045 |
| *V*_SM1RAS_ | Basal transcription rate of KRAS | 0.006 |
| *V*_SM2RAS_ | Transcription rate of KRAS regulated by EGFR and ERBB2 dimers | 0.04 |
| *V*_SEGFR_ | Translational rate of EGFR | 32 |
| *V*_SERBB2_ | Translational rate of ERBB2 | 20 |
| *V*_SRAS_ | Translational rate of KRAS | 80 |
| *k*_ASS1_ | Bimolecular rate constant for homodimerization of EGFR | 1 |
| *k*_ASS2_ | Bimolecular rate constant for homodimerization of ERBB2 | 0.000001 |
| *k*_ASS3_ | Bimolecular rate constant for heterodimerization of EGFR and ERBB2 | 0.1 |
| *k*_D_ | Dissociation rate constant of the complexes, cEGFR, cERBB2 and cERBB12 | 0.1 |
| *k*_DMEGFR_ | Degradation rate constant of EGFR mRNA | 0.083 |
| *k*_DMERBB2_ | Degradation rate constant of ERBB2 mRNA | 0.066 |
| *k*_DEGFR_ | Degradation rate constant of EGFR monomer | 0.0545 |
| *k*_DERBB2_ | Degradation rate constant of ERBB2 monomer | 0.037 |
| *k*_DCEGFR_ | Degradation rate constant of EGFR:EGFR homodimer | 0.02 |
| *k*_DCERBB2_ | Degradation rate constant of ERBB2:ERBB2 homodimer | 0.01 |
| *k*_DCERBB12_ | Degradation rate constant of EGFR:ERBB2 heterodimer | 0.01 |
| *k*_DMRAS_ | Degradation rate constant of KRAS mRNA | 0.113 |
| *k*_DRAS_ | Degradation rate constant of KRAS protein | 0.031 |
| *k*_IRAS_ | Inactivation rate constant of active KRAS protein | 10 |
| *k*_ARAS_ | Activation rate constant of inactive KRAS protein | 2 |
| *K*_A1_ | Michaelis activation constant of EGFR and ERBB2 transcription by EGFR and ERBB2 | 500 |
| *K*_A2_ | Michaelis activation constant of EGFR and ERBB2 transcription by KRAS | 250 |
| *K*_A3_ | Michaelis activation constant of KRAS activation by EGFR and ERBB2 | 100 |
| *K*_A4_ | Michaelis activation constant of KRAS transcription by EGFR and ERBB2 | 500 |

^*^The dynamic variables of the mathematical model are expressed relative to *Erbb2*/*ERBB2* mRNA expression level and are thus dimensionless. The time unit of the rate constants is inverse hour (h^-1^).

**Supplementary Table 6.** List of primary antibodies used for western blot experiments

| Antibodies | Species | Dilution | Reference |
| --- | --- | --- | --- |
| ERBB2 | Rabbit | 1/1000 | Dako (#A0485) |
| P-ERBB2^Y1248^ | Rabbit | 1/1000 | R&D Systems Europe Ltd (#AF1768) |
| EGFR | Rabbit | 1/1000 | Cell Signaling (#4267) |
| P-EGFR^Y1068^ | Rabbit | 1/1000 | Cell Signaling (#2234) |
| P-EGFR^Y1173^ | Rabbit | 1/500 | Sigma (#SAB5600079) |
| P-ERBB3^Y1289^ | Rabbit | 1/500 | Cell Signaling (#4791) |
| ERBB3 | Rabbit | 1/1000 | Cell Signaling (#12708) |
| p-ERK^T202/204^ | Rabbit | 1/2000 | Cell Signaling (#43705) |
| p-AKT^T308^ | Rabbit | 1/1000 | Cell Signaling (#2965) |
| AKT | Rabbit | 1/1000 | Cell Signaling (#9272) |
| ERK | Rabbit | 1/1000 | Cell Signaling (#9102) |
| KRAS | Rabbit | 1/200 | Homemade |
| HSC70 | Mouse | 1/2000 | Santa Cruz (#7298) |

1. **Supplementary Figure Legends**

**Supplementary Figure S1. ERBB2 is expressed in ADM and PanIN.** Immunofluorescent labeling for ErbB2 and amylase (Amy) in wild-type (WT) and ElaC^ER^ KRas^G12D^ mice in the absence of inflammation and after induction of acute (W1) or chronic (W3 and W9) pancreatitis. Data are the same as those in Fig. 1 but the red and green channels were separated to better visualize each labeling. Scale bars = 50 μm.

**Supplementary Figure S2. *Erbb2* deletion does not affect the expression of metaplastic markers.** Immunofluorescent labeling of ERBB2, CK19 and SOX9 in WT and ElaC^ER^ Erbb2^KO^ mice treated 3 weeks with cerulein (W3) and in ElaC^ER^ KRas^G12D^ and ElaC^ER^ Kras^G12D^ Erbb2^KO^ mice treated 1 week with cerulein (W1). Efficient deletion of ERBB2 is detected in ElaC^ER^ Erbb2^KO^ and ElaC^ER^ Kras^G12D^ Erbb2^KO^ mice. CK19 and SOX9 are detected in ductal cells as well as in duct-like cells and in PanIN. Scale bars = 50 μm.

**Supplementary Figure S3. *Erbb2* deletion is efficient but heterogenous.** The number of ERBB2- and EGFR-positive cells (approximately 600 cells counted for both genotypes) were quantified manually in PanIN of 9-weeks cerulein-treated ElaC^ER^ Kras^G12D^ and ElaC^ER^ Erbb2^KO^ Kras^G12D^ mice. The illustration of cells considered positive or negative for both markers is visible below the quantification. (A) The number of ERBB2-positive cells in PanIN is reduced by about 6-fold in ElaC^ER^ Erbb2^KO^ Kras^G12D^ mice compared to ElaC^ER^ Kras^G12D^ mice treated for 9 weeks with cerulein (W9). Percentages are mean +/- SD. (B) The number of EGFR-positive cells is similar in ElaC^ER^ Erbb2^KO^ Kras^G12D^ mice and ElaC^ER^ Kras^G12D^ mice. Percentages are mean +/- SD; ns, p>0.05. (C) In ElaC^ER^ Kras^G12D^ ROSA26R^YFP^ mice, the majority of PanIN derived from acinar cells (GFP positives) express ERBB2 (white asterisks). In ElaC^ER^ Erbb2^KO^ Kras^G12D^ ROSA26R^YFP^ mice, the majority of PanIN derived from acinar cells (GFP positives) no longer express ERBB2 (yellow asterisks) with the exception of a few PanIN positive for ERBB2 (white asterisk). Ductal cells (GFP negatives) express ERBB2 in both mice (inset). Scale bars = 50 μm.

**Supplementary Figure S4. *Erbb2* deletion does not affect collagen deposition or immune cell infiltration.** (A) Sirius Red and Fast Green staining of WT, ElaC^ER^ Erbb2^KO^, ElaC^ER^ Kras^G12D^ and ElaC^ER^ Erbb2^KO^ Kras^G12D^ mice after 9 weeks of cerulein treatment (W9). Collagen deposits were present in all mice, with significantly more collagen in ElaC^ER^ Kras^G12D^ and ElaC^ER^ Kras^G12D^ Erbb2^KO^ mice. Scale bars = 50 μm. (B). Immunohistochemical staining for CD45 after 3 weeks of cerulein treatment (W3). No difference in the proportions of CD45-positive cells is detected between WT and ElaC^ER^ Erbb2^KO^ mice, and between ElaC^ER^ Kras^G12D^ and ElaC^ER^ Kras^G12D^ Erbb2^KO^ mice. Scale bars = 50 μm.

**Supplementary Figure S5. Expression and activity of other ERBB members is not affected by *Erbb2* deletion.** Immunofluorescent labeling for EGFR, P-EGFR^Y845^, ERBB3, and Amylase (Amy) in WT and ElaC^ER^ Erbb2^KO^ mice treated for 1 week with cerulein (W1). EGFR and ERBB3 are similarly expressed or activated in ADM of WT and ElaC^ER^ Erbb2^KO^ pancreas (white arrows). Scale bars = 50 μm.

**Supplementary Figure S6. *Erbb2* deletion does not affect expression and activity of other ERBB members.** Immunofluorescent labeling for EGFR, P-EGFR^Y845^, ERBB3, and Amylase in WT and ElaC^ER^ Erbb2^KO^ mice treated 3 weeks with cerulein (W3) and in ElaC^ER^ Kras^G12D^ and ElaC^ER^ Kras^G12D^ Erbb2^KO^ mice treated 1 week with cerulein (W1). EGFR and ERBB3 are similarly expressed or activated in ADM of WT and ElaC^ER^ Erbb2^KO^ pancreas (white arrows) and in PanIN present in ElaC^ER^ Kras^G12D^ and ElaC^ER^ Kras^G12D^ Erbb2^KO^ pancreas. DAPI staining visualizes cell nuclei. Scale bars = 50 μm.

**Supplementary Figure S7. ERBB signaling effectors are not affected by *Erbb2* loss.** Immunohistochemical staining for P-ERK (T202/204) and P-AKT (T308) after 1 week of cerulein treatment (W1). P-ERK and P-AKT stain a small proportion of ADM in WT and ElaC^ER^ Erbb2^KO^ mice whereas large numbers of PanIN are stained in ElaC^ER^ Kras^G12D^ and ElaC^ER^ Kras^G12D^ Erbb2^KO^ mice. Scale bars = 50 μm.

**Supplementary Figure S8. Expression of ERBB receptors and their ligands.** (A) Human single-cell mRNA expression levels of EGFR and two of these ligands, EGF and TGFα (black bars), of ERBB3 and its ligands, NRG1 and NRG2 (blue bars), of ERBB2 (grey bars), SOX9 (red bars) and AMY2A (green bars) in acinar cells (n = 24), in ductal cells (n = 28), in alpha (n = 239), beta (n = 264), delta (n = 25), gamma/PP (n = 18) and stellate cells (n = 19). Data are from [14]. (B) mRNA expression of EGFR, ERBB2 and ERBB3 and the ligands EGF, TGFα, NRG1 and NRG2 in the PDAC cohort from TCGA (n = 178). ERBB2 expression was arbitrarily set to 1. (C) mRNA expression levels in WT mice in the absence (blue bars) or in the presence of 1-week cerulein treatment (red bars) (n = 7). Erbb2 expression was arbitrarily set to 100. mRNA expressions are mean +/- SEM. (D) Western blots for P-ERBB3^Y1289^ and ERBB3 in WT, ElaC^ER^ Erbb2^KO^, ElaC^ER^ Kras^G12D^ and ElaC^ER^ Kras^G12D^ Erbb2^KO^ mice treated for 1 week with cerulein (W1). ERBB3 is not phosphorylated in all animals. C+: human pancreatic cancer organoid with active ERBB3 signaling. Phosphorylated and non-phosphorylated forms of ERBB3 were detected on the same blot. Consequently, HSC70 loading controls were the same. Blots were cropped and full-length blots are avaible in Supplementary Fig. S12.

**Supplementary Figure S9.** **Robustness of the predicted impact of *ERBB2* on ERBB signaling activity.** Steady-state levels of ERBB signaling activity (detailed definition in section 1) are shown as a function of *ERBB2* mRNA levels in basal parameter conditions (black) and following a ten-fold increase (red) or decrease (green) of (*A*) *T*_ERBB2_, (*B*) *T*_RAS_, (*C*) *V*_SEGFR_, (*D*) *V*_SERBB2_, (*E*) *V*_SRAS_, (*F*) *k*_ASS1_, (*G*) *k*_ASS2_, (*H*) *k*_ASS3_, (*I*) *K*_A1_, (*J*) *K*_A2_, (*K*) *K*_A3_, (*L*) *K*_A4_, (*M*) *k*_ARAS_, (*N*) *k*_IRAS_, (*O*) *k*_DCEGFR_, (*P*) *k*_DCERBB2_, (*Q*) *k*_DCERBB12_. Other parameter values are as in Supplementary Table 5.

**Supplementary Figure S10.** ***EGFR* is required for ERBB signaling activity.** Steady-state levels of ERBB signaling activity are shown as a function of *EGFR* mRNA levels in basal parameter conditions (black) and following a ten-fold increase (red) or decrease (green) of (*A*) *T*_ERBB2_, (*B*) *T*_RAS_, (*C*) *V*_SEGFR_, (*D*) *V*_SERBB2_, (*E*) *V*_SRAS_, (*F*) *k*_ASS1_, (*G*) *k*_ASS2_, (*H*) *k*_ASS3_, (*I*) *K*_A1_, (*J*) *K*_A2_, (*K*) *K*_A3_, (*L*) *K*_A4_, (*M*) *k*_ARAS_, (*N*) *k*_IRAS_, (*O*) *k*_DCEGFR_, (*P*) *k*_DCERBB2_, (*Q*) *k*_DCERBB12_. Other parameter values are as in Supplementary Table 5.

**Supplementary Figure S11.** **ERBB2 overexpression partially impacts ERBB signaling activity.** Representative immunoblots of proteins extracted from MiaPaCa-2 cells infected with empty or mErbb2 lentiviruses. Levels of ERBB2, EGFR and their phosphorylated forms, as well as the KRAS-GTP/KRAS ratio, were quantified; P-ERBB2^Y1248^, ERBB2 and P-EGFR^Y1068^ increased significantly after mErbb2 lentiviral infection whereas P-EGFR^Y1173^ and KRAS-GTP/KRAS ratio do not reach significance. Fold inductions are mentioned as mean +/- SD; ns, p>0.05. Phosphorylated and non-phosphorylated forms of ERBB2 and EGFR were detected on the same blots. Consequently, HSC70 loading controls were the same. Blots were cropped and full-length blots are avaible in Supplementary Figures S16 to S18.

**Supplementary Figure S12.** **Full-length blots of P-ERBB3^Y1289^, ERBB3 and HSC70 related to Figure S8D.** The lanes of interest have been framed in black.

**Supplementary Figure S13.** **Full-length blots of P-ERBB2^Y1248^, ERBB2 and HSC70 related to Figure 7.** The lanes of interest have been framed in black. The others are no related to this experiment.

**Supplementary Figure S14.** **Full-length blots of P-EGFR^Y1068^, EGFR and HSC70 related to Figure 7.** The lanes of interest have been framed in black. The others are no related to this experiment.

**Supplementary Figure S15.** **Full-length blots of P-EGFR^Y1173^, EGFR and HSC70 related to Figure 7.** The lanes of interest have been framed in black. The others are no related to this experiment.

**Supplementary Figure S16.** **Full-length blots of P-ERBB2^Y1248^, ERBB2 and HSC70 related to Figure S11.** The lanes of interest have been framed in black. The others are no related to this experiment.

**Supplementary Figure S17.** **Full-length blots of P-EGFR^Y1068^, EGFR and HSC70 related to Figure S11.** The lanes of interest have been framed in black. The others are no related to this experiment.

**Supplementary Figure S18.** **Full-length blots of P-EGFR^Y1173^, EGFR and HSC70 related to Figure S11.** The lanes of interest have been framed in black. The others are no related to this experiment.

1. **Supplementary Figures**


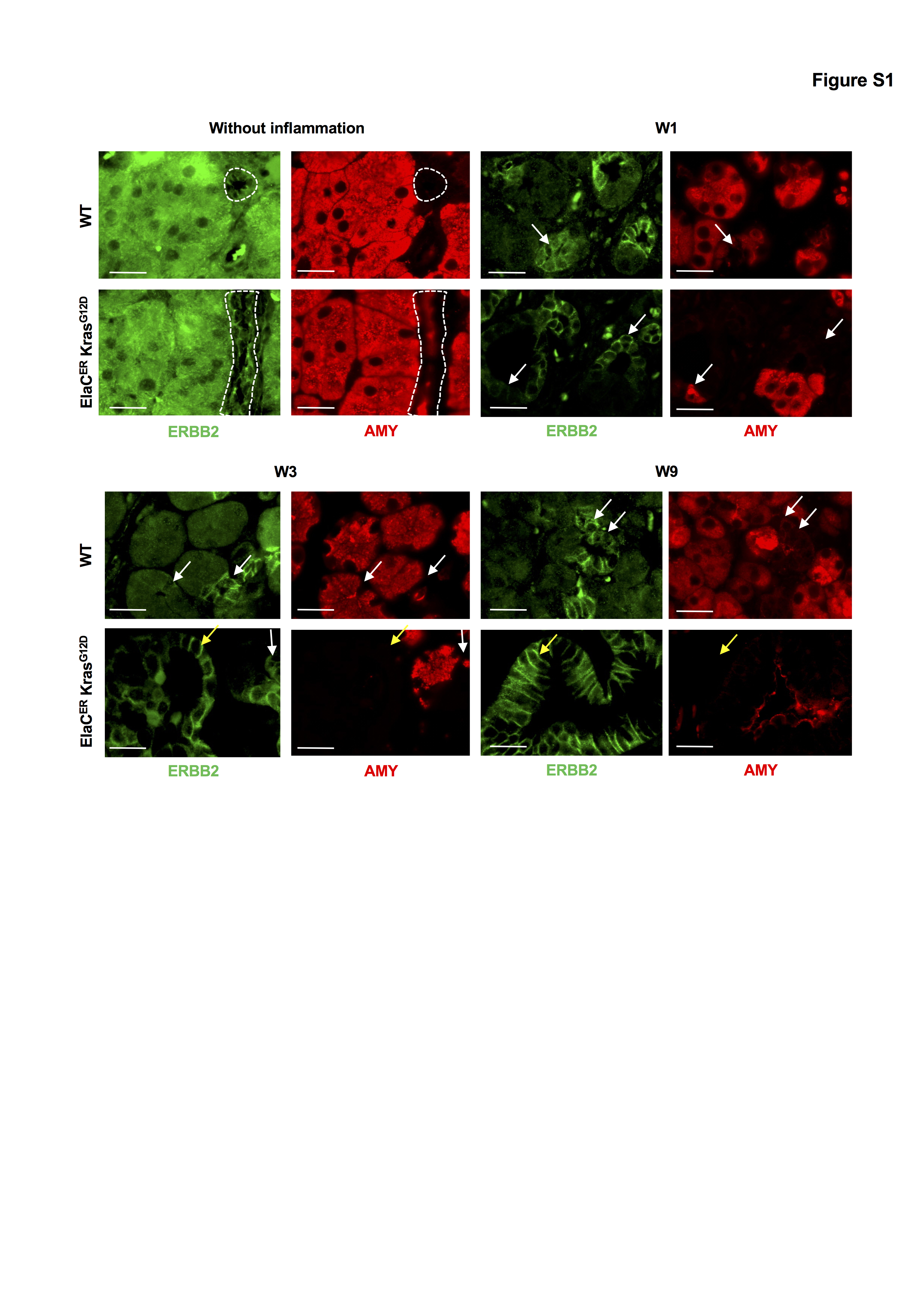


**Supplementary Figure S1**

**
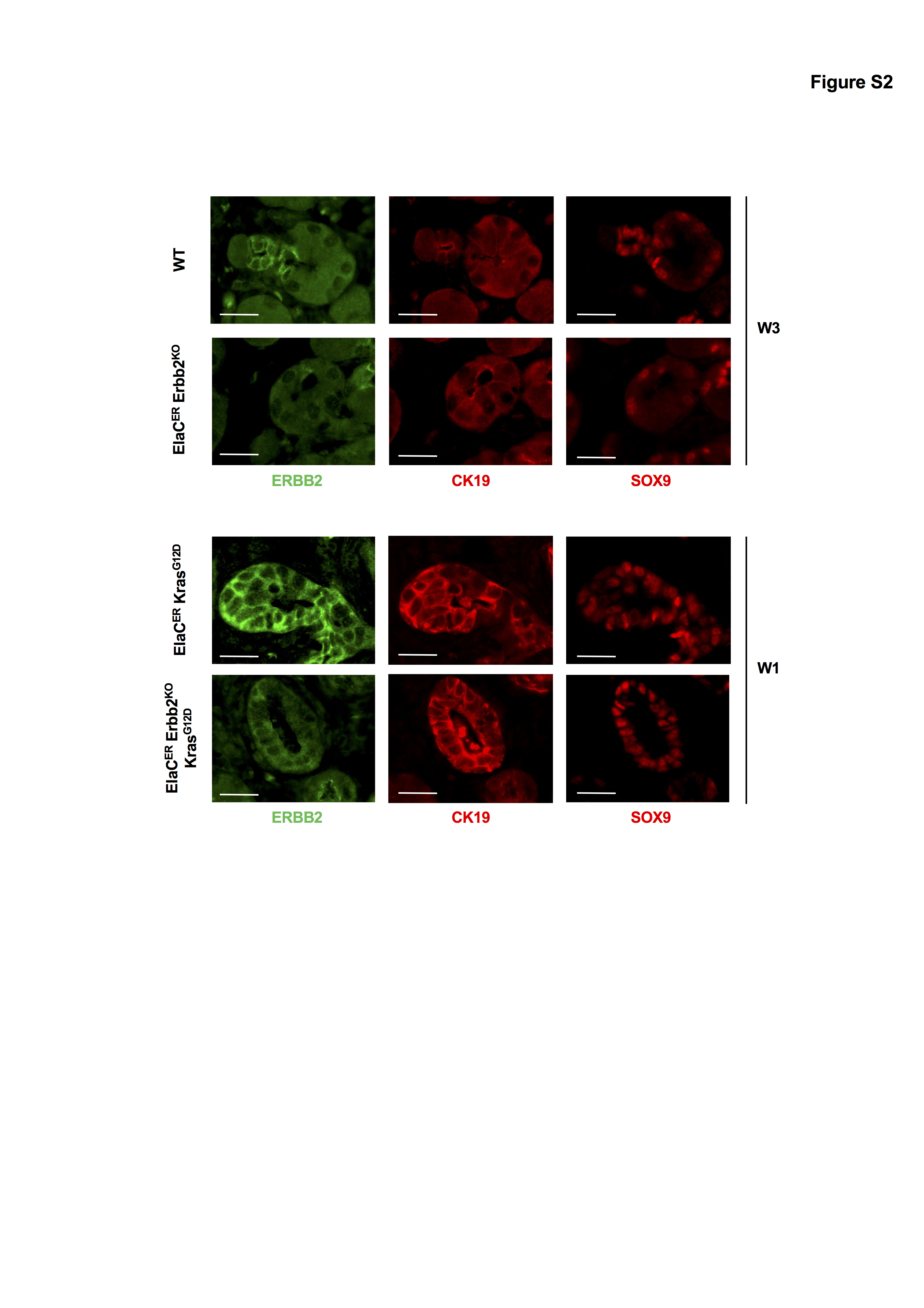
**

**Supplementary Figure S2**

**
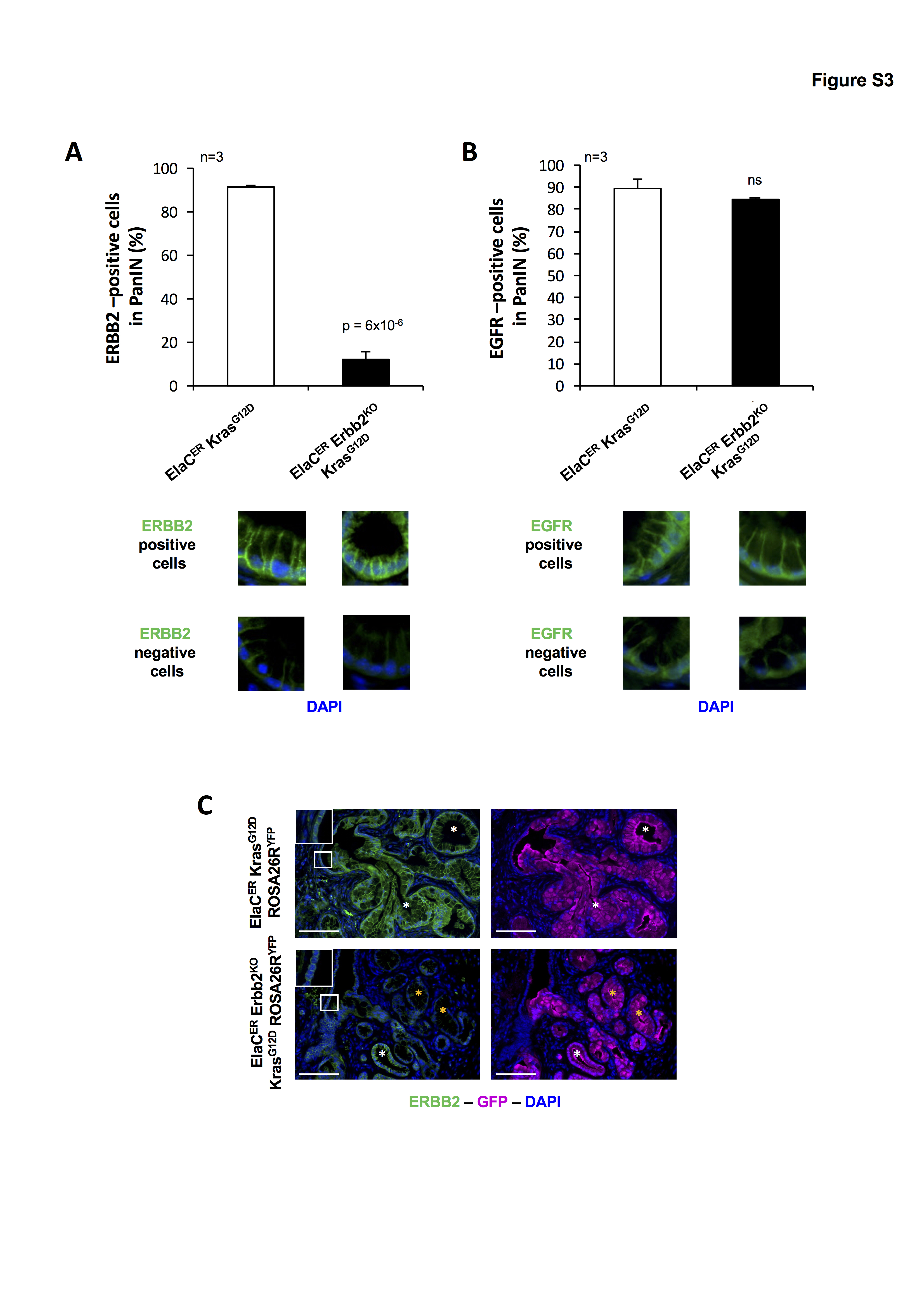
**

**Supplementary Figure S3**

**
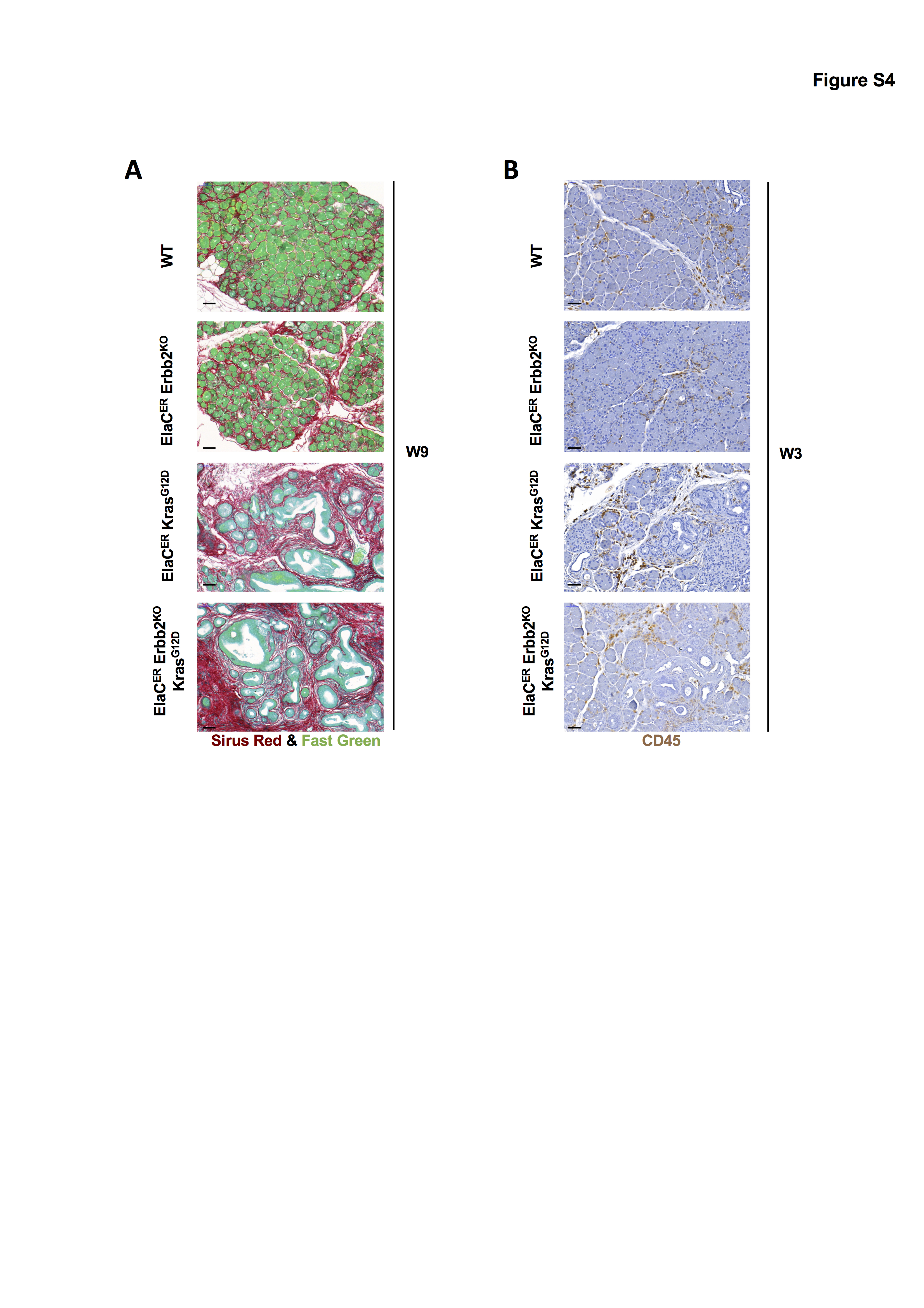
**

**Supplementary Figure S4**

**
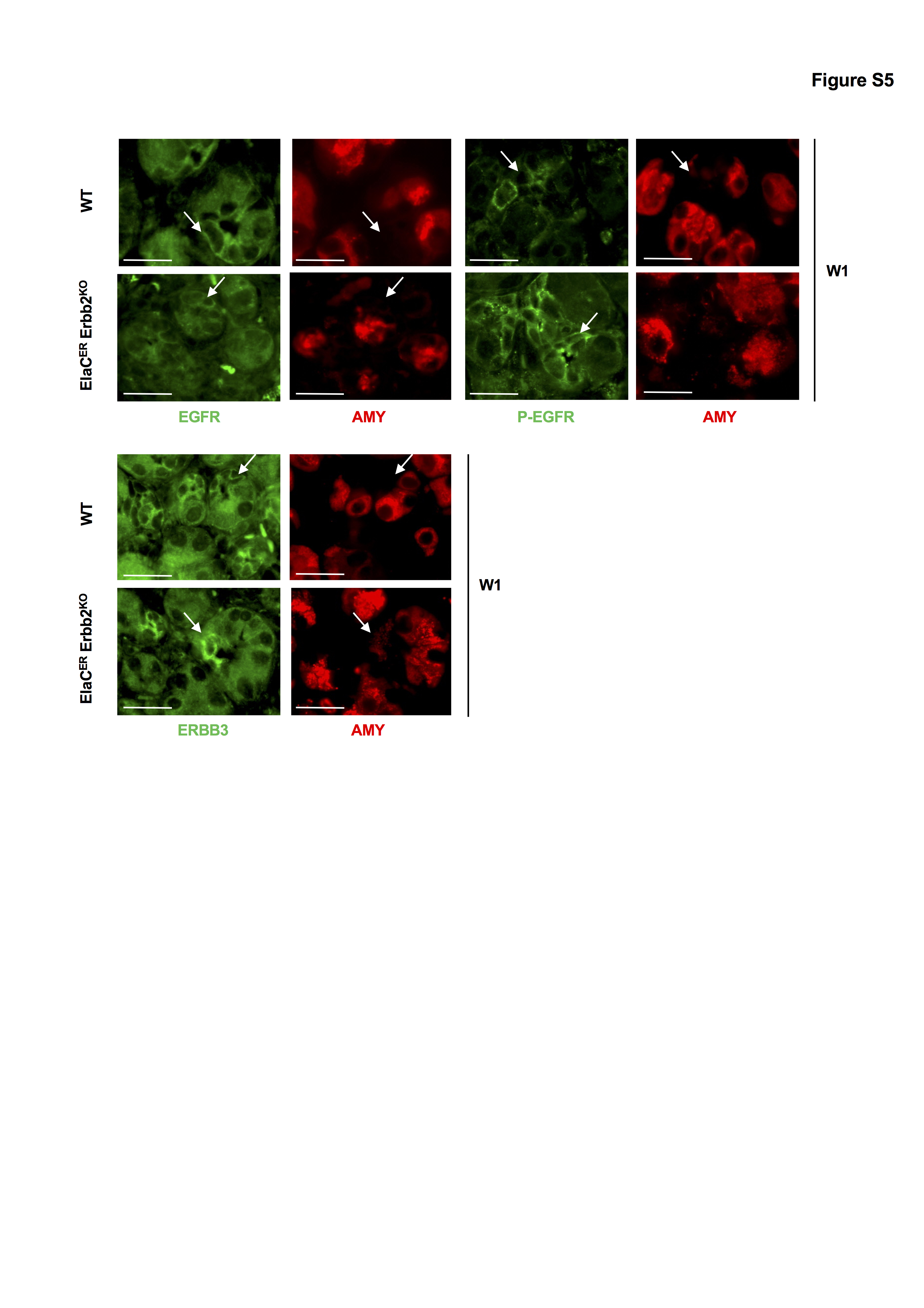
**

**Supplementary Figure S5**

**
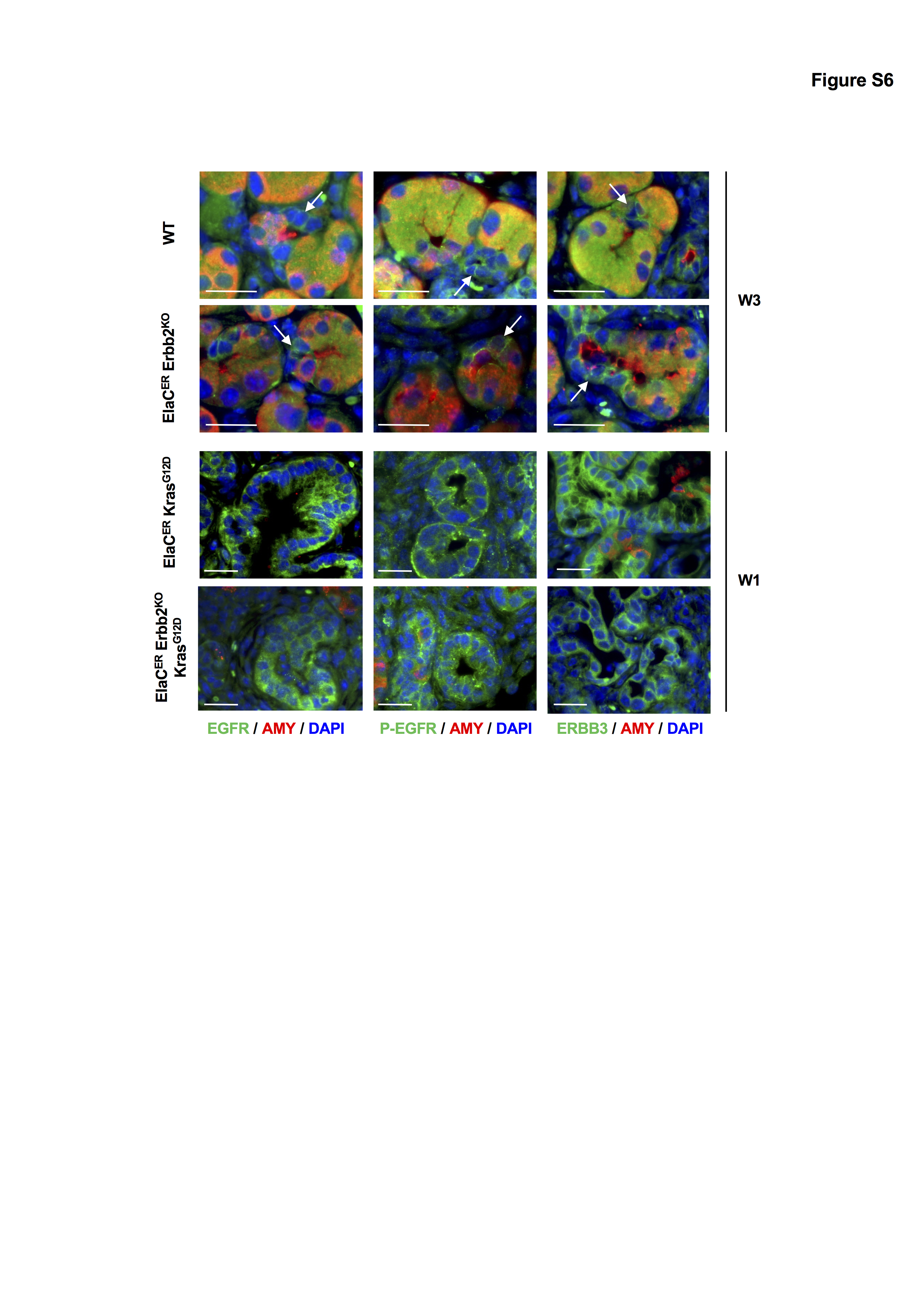
**

**Supplementary Figure S6**

**
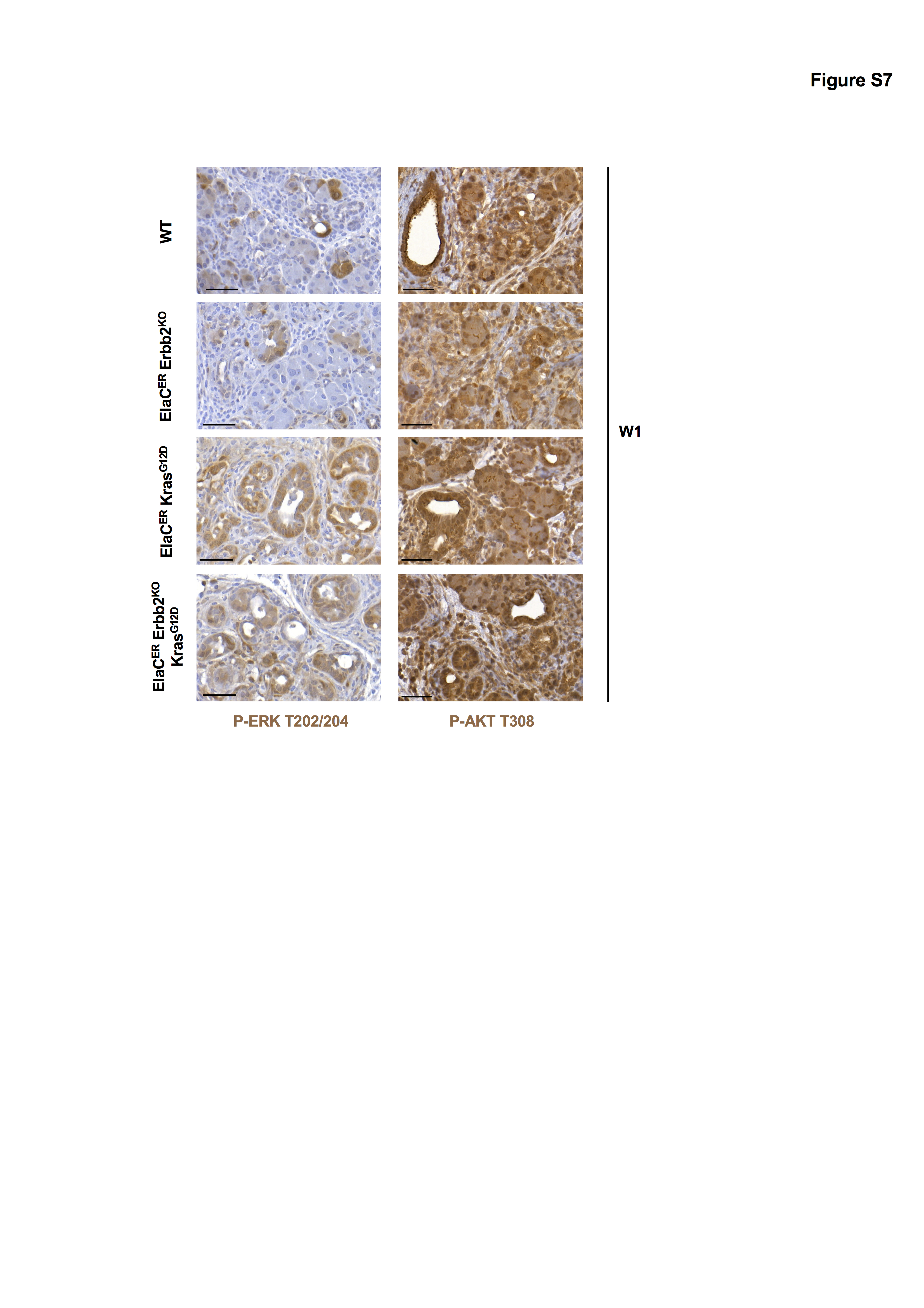
**

**Supplementary Figure S7**

**
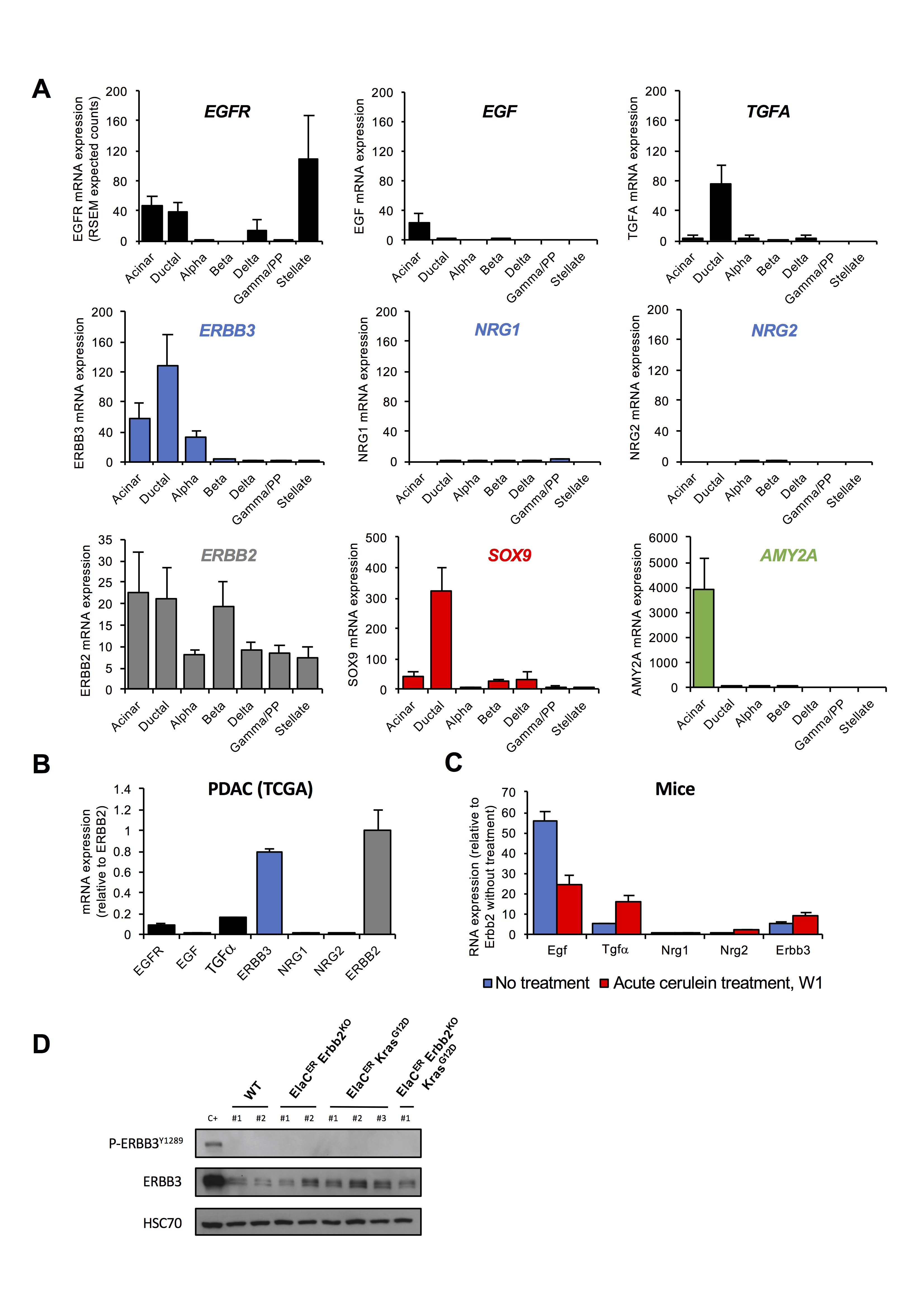
**

**Supplementary Figure S8**

**
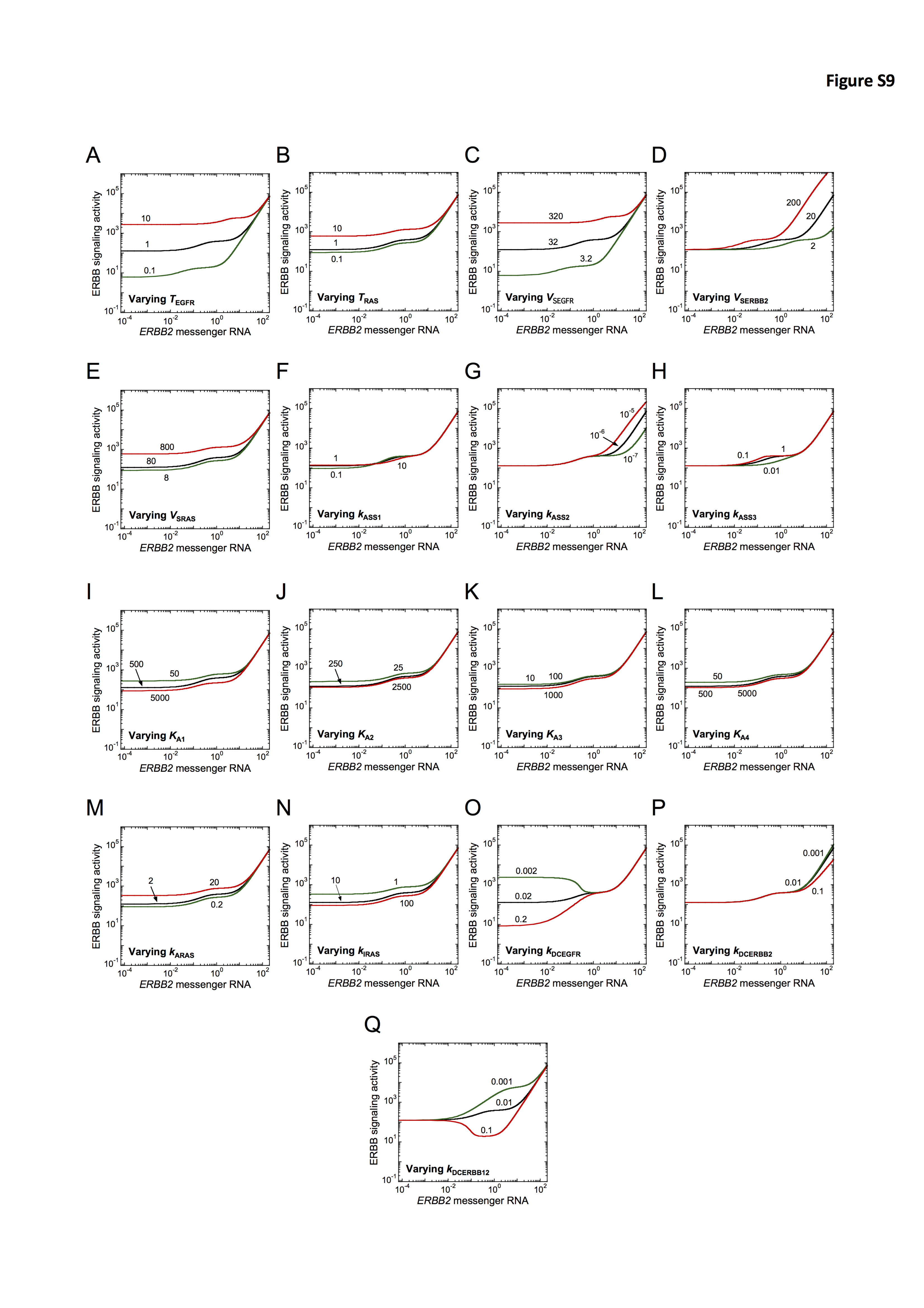
**

**Supplementary Figure S9**

**
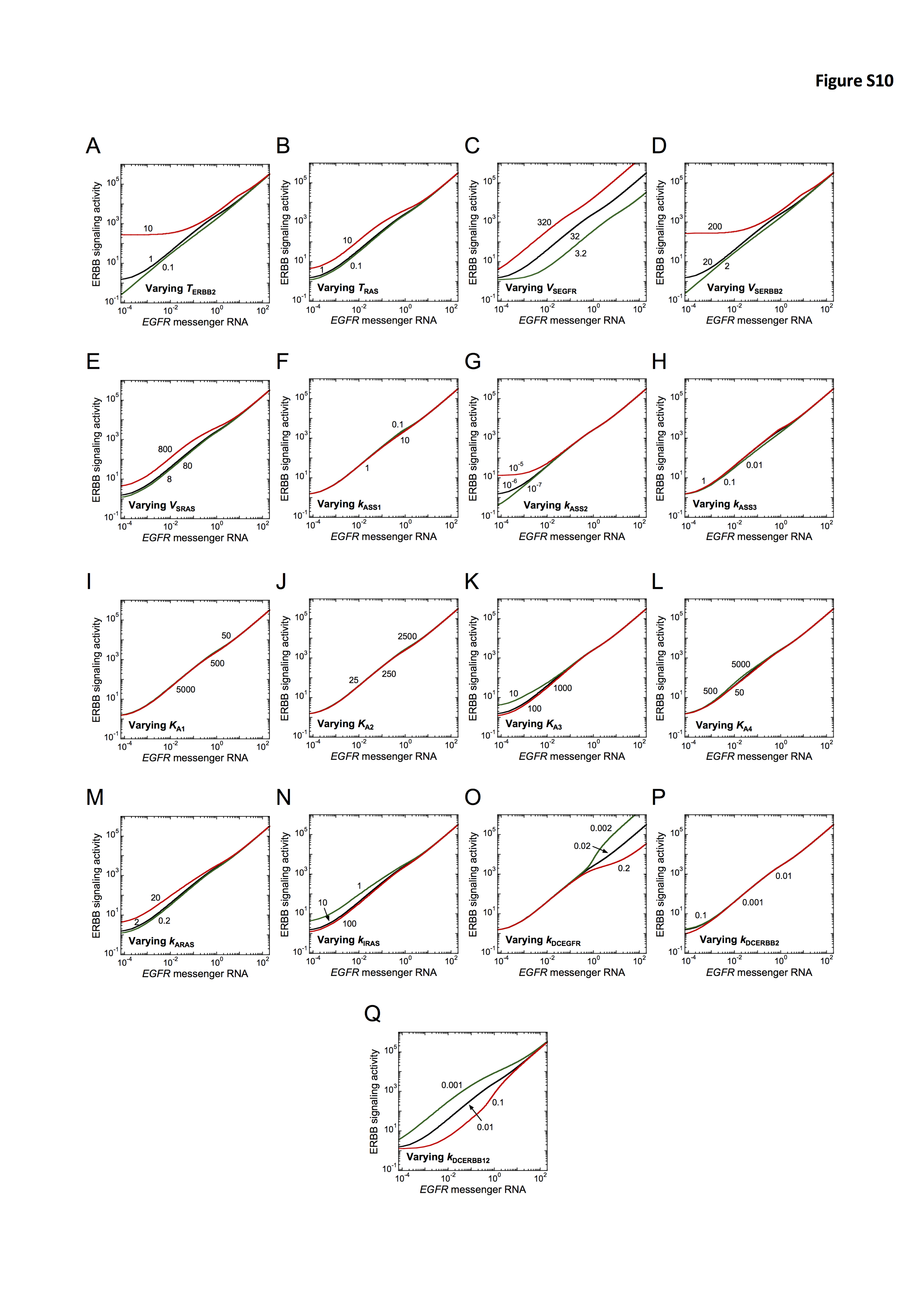
**

**Supplementary Figure S10**

**
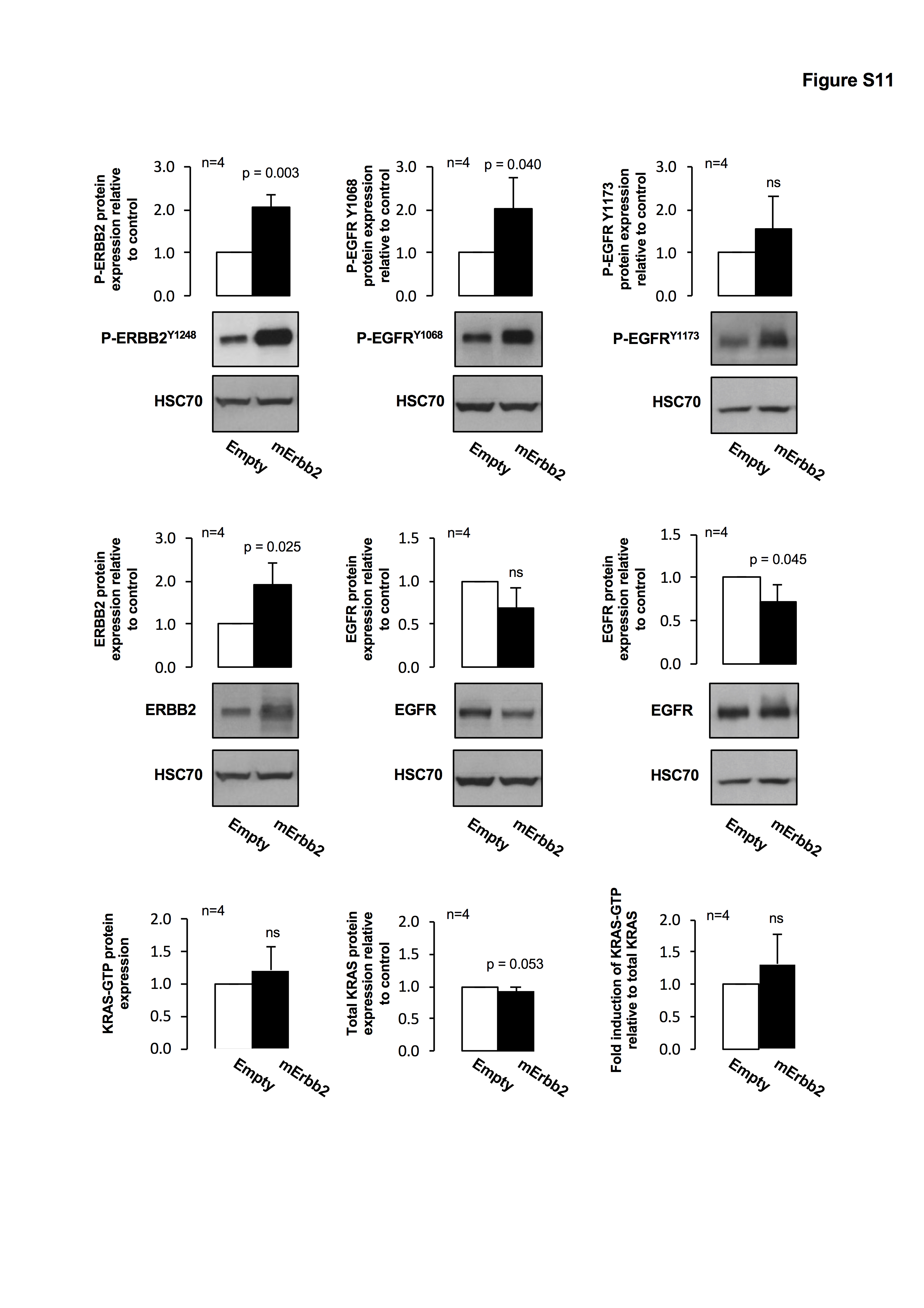
**

**Supplementary Figure S11**

**
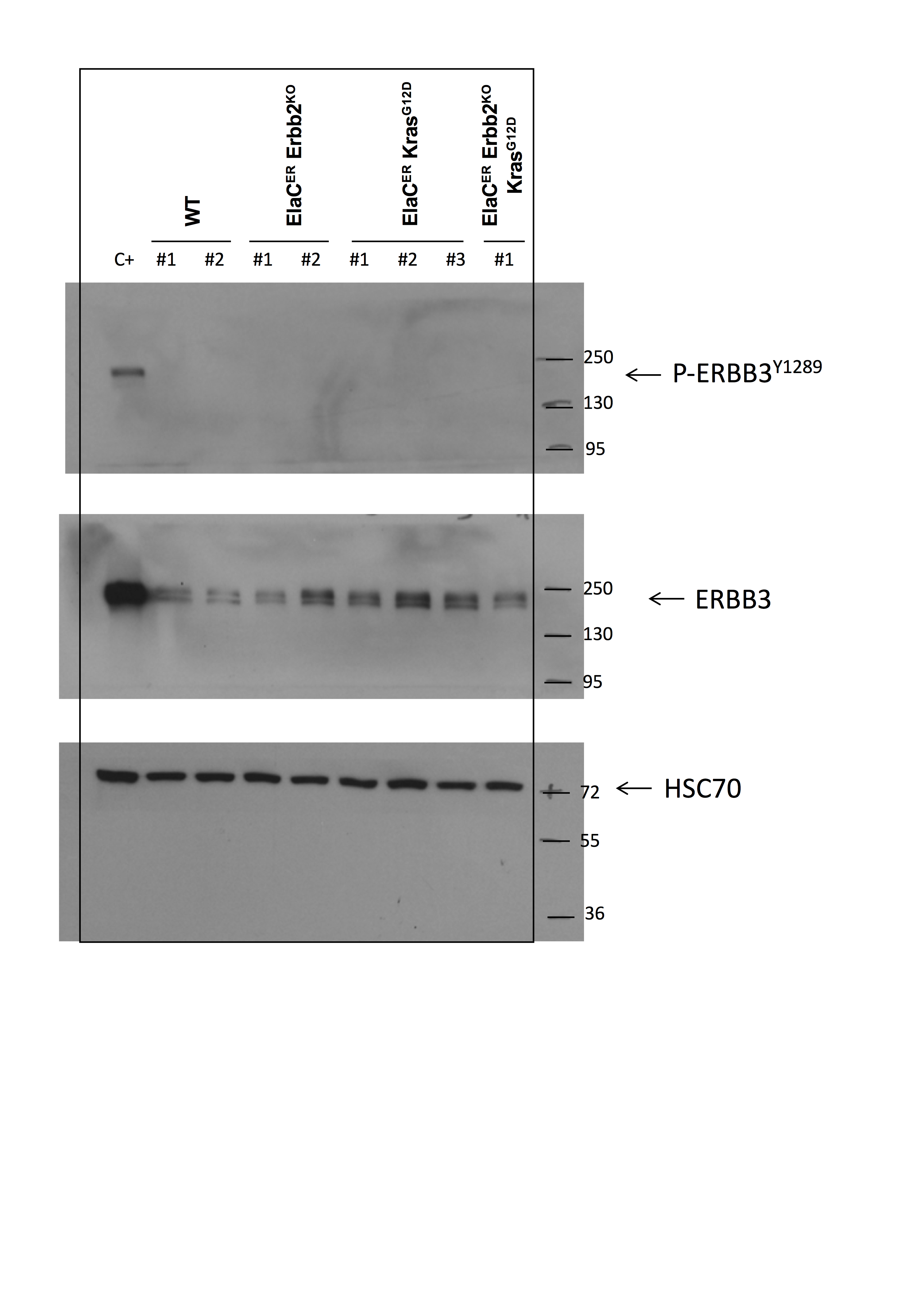
**

**Supplementary Figure S12**

**
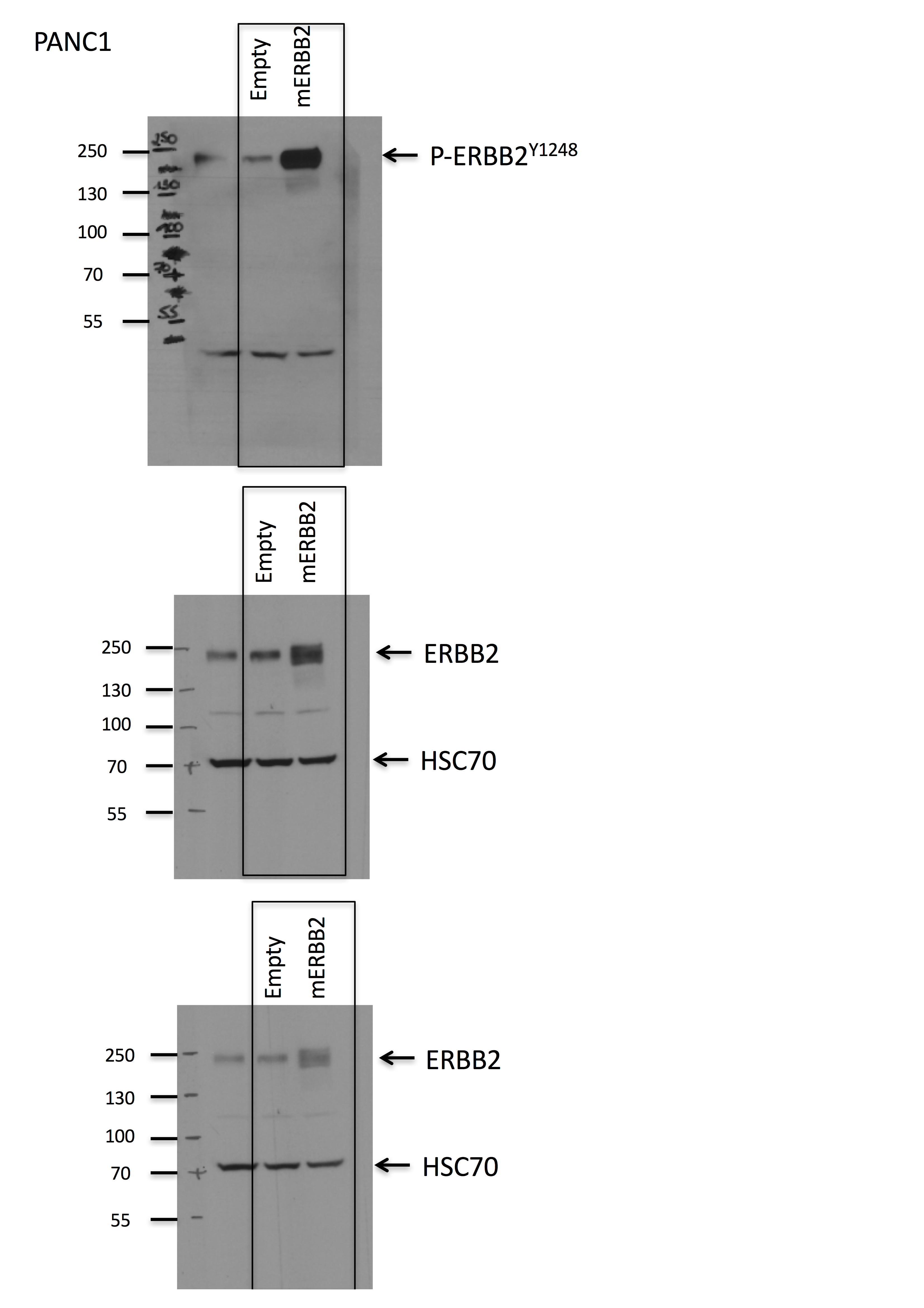
**

**Supplementary Figure S13**

**
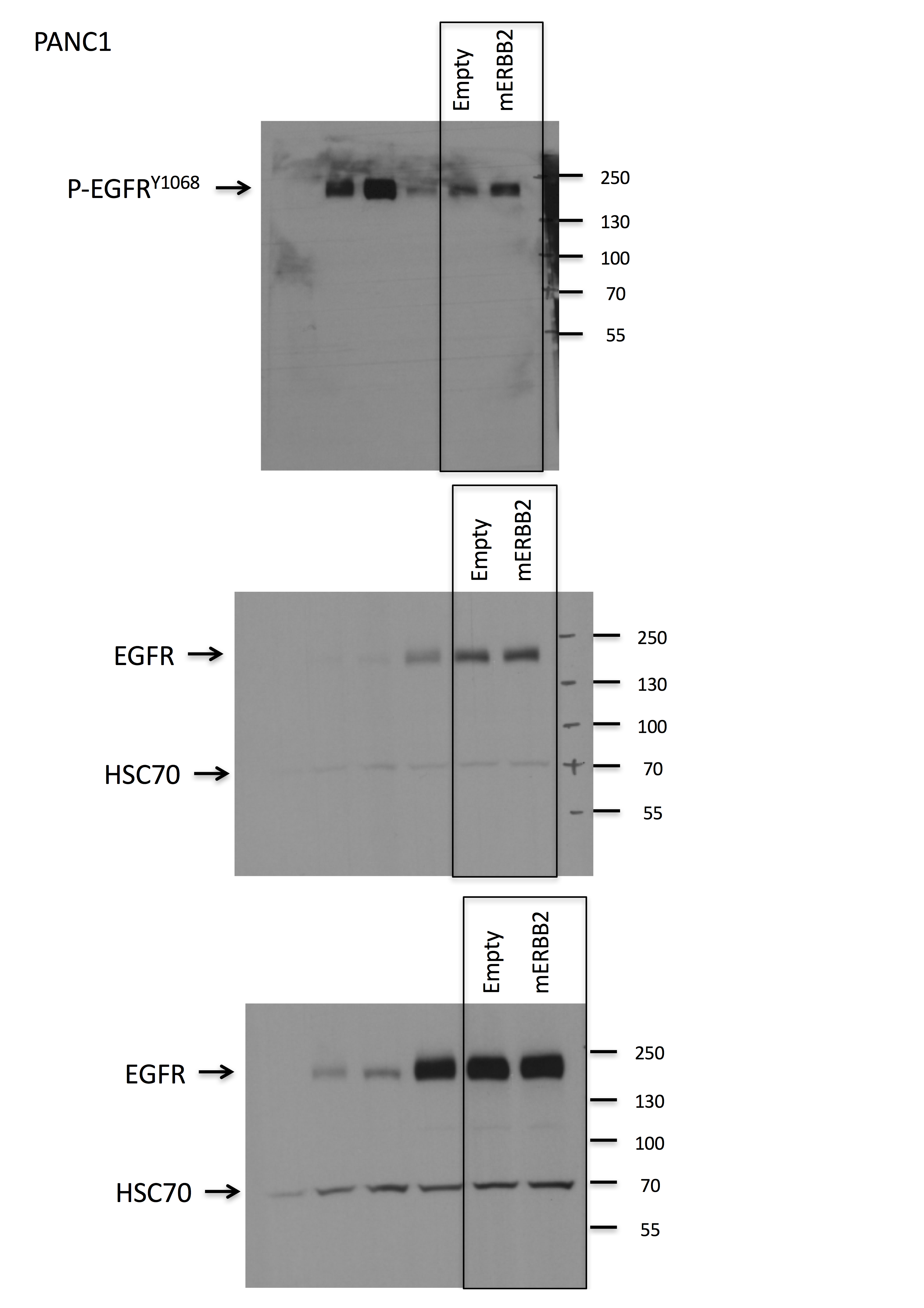
**

**Supplementary Figure S14**

**
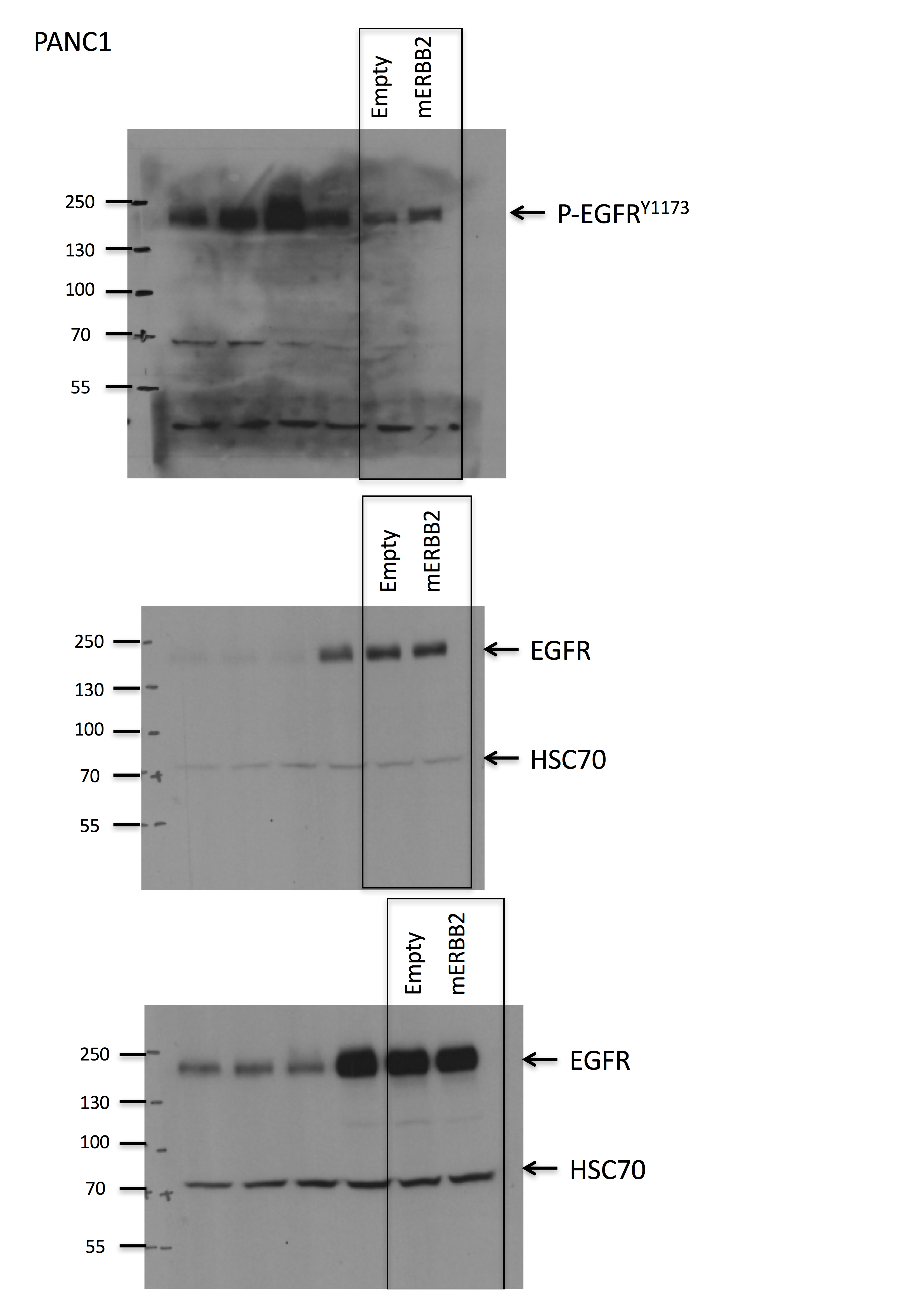
**

**Supplementary Figure S15**

**
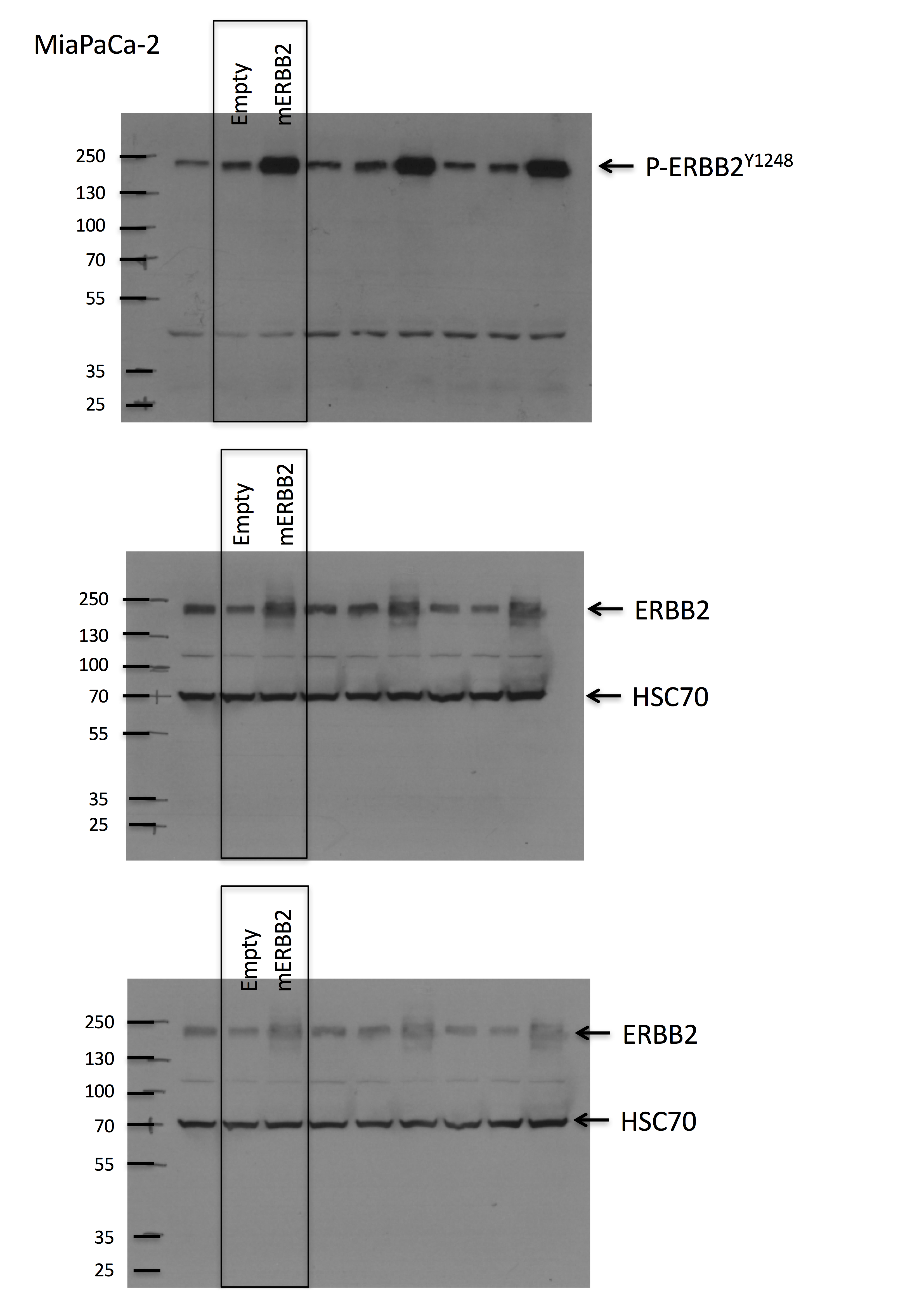
**

**Supplementary Figure S16**

**
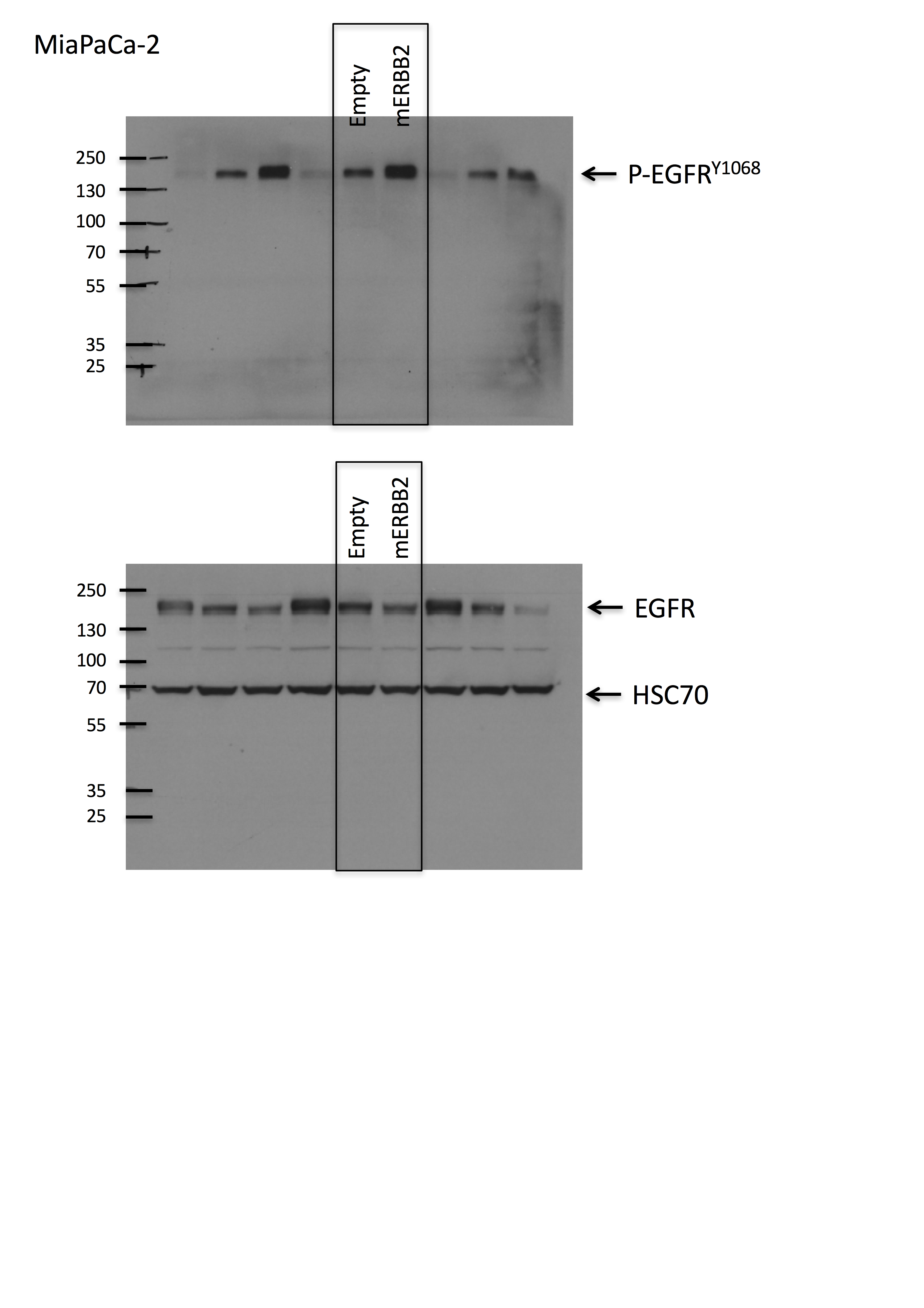
**

**Supplementary Figure S17**

**
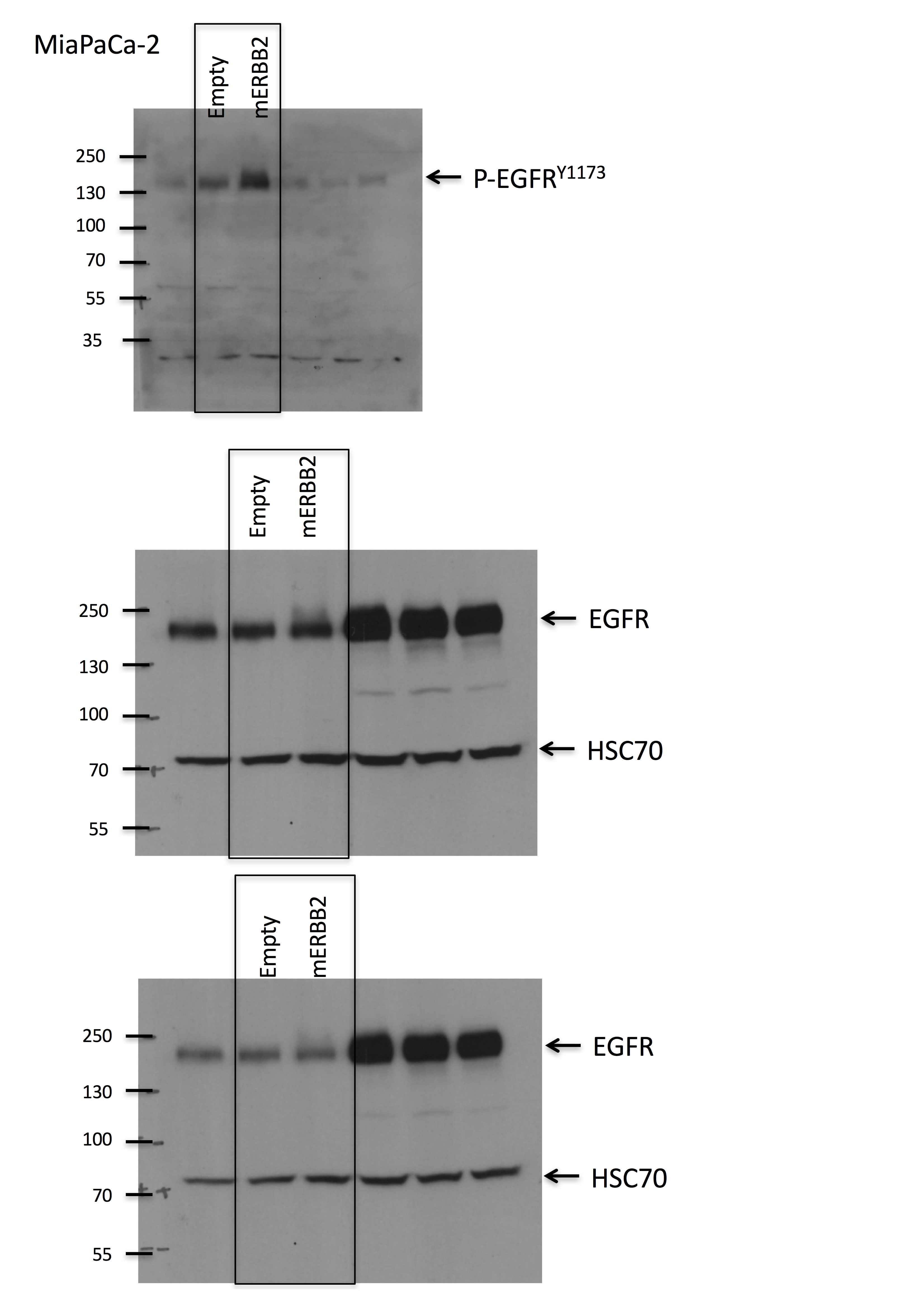
**

**Supplementary Figure S18**

1. **Supplementary references**

1 Ardito CM, Gruner BM, Takeuchi KK, Lubeseder-Martellato C, Teichmann N, Mazur PK *et al*. EGF receptor is required for KRAS-induced pancreatic tumorigenesis. *Cancer Cell* 2012; 22: 304-317.

2 Algul H, Wagner M, Lesina M, Schmid RM. Overexpression of ErbB2 in the exocrine pancreas induces an inflammatory response but not increased proliferation. *Int J Cancer* 2007; 121: 1410-1416.

3 Wagner M, Greten FR, Weber CK, Koschnick S, Mattfeldt T, Deppert W *et al*. A murine tumor progression model for pancreatic cancer recapitulating the genetic alterations of the human disease. *Genes Dev* 2001; 15: 286-293.

4 Grimont A, Pinho AV, Cowley MJ, Augereau C, Mawson A, Giry-Laterriere M *et al*. SOX9 regulates ERBB signalling in pancreatic cancer development. *Gut* 2015; 64: 1790-1799.

5 Diersch S, Wirth M, Schneeweis C, Jors S, Geisler F, Siveke JT *et al*. Kras(G12D) induces EGFR-MYC cross signaling in murine primary pancreatic ductal epithelial cells. *Oncogene* 2016; 35: 3880-3886.

6 Navas C, Hernandez-Porras I, Schuhmacher AJ, Sibilia M, Guerra C, Barbacid M. EGF receptor signaling is essential for k-ras oncogene-driven pancreatic ductal adenocarcinoma. *Cancer Cell* 2012; 22: 318-330.

7 Hingorani SR, Wang L, Multani AS, Combs C, Deramaudt TB, Hruban RH *et al*. Trp53R172H and KrasG12D cooperate to promote chromosomal instability and widely metastatic pancreatic ductal adenocarcinoma in mice. *Cancer Cell* 2005; 7: 469-483.

8 Zhu L, Shi G, Schmidt CM, Hruban RH, Konieczny SF. Acinar cells contribute to the molecular heterogeneity of pancreatic intraepithelial neoplasia. *Am J Pathol* 2007; 171: 263-273.

9 Aguirre AJ, Bardeesy N, Sinha M, Lopez L, Tuveson DA, Horner J *et al*. Activated Kras and Ink4a/Arf deficiency cooperate to produce metastatic pancreatic ductal adenocarcinoma. *Genes Dev* 2003; 17: 3112-3126.

10 Zhao S, Wang Y, Cao L, Ouellette MM, Freeman JW. Expression of oncogenic K-ras and loss of Smad4 cooperate to induce the expression of EGFR and to promote invasion of immortalized human pancreas ductal cells. *Int J Cancer* 2010; 127: 2076-2087.

11 Siveke JT, Einwachter H, Sipos B, Lubeseder-Martellato C, Kloppel G, Schmid RM. Concomitant pancreatic activation of Kras(G12D) and Tgfa results in cystic papillary neoplasms reminiscent of human IPMN. *Cancer Cell* 2007; 12: 266-279.

12 Garrett TP, McKern NM, Lou M, Elleman TC, Adams TE, Lovrecz GO *et al*. The crystal structure of a truncated ErbB2 ectodomain reveals an active conformation, poised to interact with other ErbB receptors. *Mol Cell* 2003; 11: 495-505.

13 Schwanhausser B, Busse D, Li N, Dittmar G, Schuchhardt J, Wolf J *et al*. Global quantification of mammalian gene expression control. *Nature* 2011; 473: 337-342.

14 Segerstolpe A, Palasantza A, Eliasson P, Andersson EM, Andreasson AC, Sun X *et al*. Single-Cell Transcriptome Profiling of Human Pancreatic Islets in Health and Type 2 Diabetes. *Cell Metab* 2016; 24: 593-607.
